# Thermally Driven Dynamic Behaviors in Polymeric Vesicles

**DOI:** 10.1002/smll.202411220

**Published:** 2025-03-05

**Authors:** Matthew E. Allen, Yeyang Sun, Chi Long Chan, Miguel Paez‐Perez, Oscar Ces, Yuval Elani, Claudia Contini

**Affiliations:** ^1^ Department of Chemistry Molecular Sciences Research Hub Imperial College London London W12 0BZ UK; ^2^ Institute of Chemical Biology Molecular Sciences Research Hub Imperial College London London W12 0BZ UK; ^3^ Department of Chemical Engineering Imperial College London South Kensington London SW7 2AZ UK; ^4^ fabriCELL, Molecular Sciences Research Hub Imperial College London London W12 0BZ UK; ^5^ Department of Life Sciences Imperial College London South Kensington London SW7 2AZ UK

**Keywords:** biomimicry, polymersomes, self‐assembly, synthetic cells, thermally responsive

## Abstract

Stimuli‐responsive polymeric vesicles offer a versatile platform for mimicking dynamic cell‐like behaviors for synthetic cell applications. In this study, thermally responsive polymeric droplets derived from poly(ethylene oxide)‐poly(butylene oxide) (PEO‐PBO) polymersomes, aiming to create synthetic cell models that mimic key biological functions are developed. Upon heating, the nanoscale vesicles undergo fusion, transforming into sponge‐like microscale droplets enriched with membrane features. By modulating the temperature, these droplets display dynamic properties such as contractility, temperature‐induced fusion, and cargo trapping, including small molecules and bacteria, thereby demonstrating their ability to dynamically interface with biological entities. The findings demonstrate the potential of our sponge‐like droplets in synthetic cell applications, contributing to the understanding of PEO‐PBO polymersomes’ unique characteristics, expanding the capabilities of synthetic cell structures, and representing an exciting possibility for advancing soft matter engineering to cell‐like behaviors.

## Graphical abstract

Synthetic cells aim to mimic biological form, function, and behavior. In this article, the thermoresponsive properties of the polymer poly(ethylene oxide)‐poly(butylene oxide) are leveraged to create a range of polymeric synthetic cell systems on the nanoscale and microscale which possess a range of biomimetic properties, including cargo release, thermal contractility, membrane fusion, and material capture.

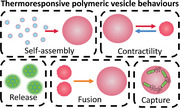

## 1 Introduction

Recent advancements in synthetic cell technology have opened up a world of possibilities for creating microsystems that can mimic essential biological behaviors and functions.^[^ ^1^, ^2^, ^3^, ^4^ ^]^ These include motility,^[^ ^5^ ^]^ compartmentalization,^[^ ^6^ ^]^ growth,^[^ ^7^ ^]^ division,^[^ ^8^ ^]^ and energy transduction.^[^ ^9^ ^]^ Synthetic cells have found applications in a wide range of fields including as therapeutic delivery vehicles,^[^ ^10^ ^]^ as biosensensors,^[^ ^11^ ^]^ and as microreactors.^[^ ^12^ ^]^ However, as synthetic cell technology advances rapidly, the need for mimicry of more complex behaviors becomes increasingly essential.

To address this challenge, polymersomes are now being explored as a versatile chassis for synthetic cells. Polymersomes, polymeric vesicles that can be created from block copolymers,^[^ ^13^, ^14^, ^15^ ^]^ offer a versatile platform for creating synthetic cells with tailored properties and tunable functionalities, providing an alternative to the sometimes complex formulations required in lipid‐based synthetic cells. To date, polymersomes have enabled light‐activated permeability of small molecules across polymer membranes,^[^ ^16^ ^]^ spatiotemporal control of signal‐driven enzymatic reactions.^[^ ^17^ ^]^ and chemotactic motility.^[^ ^18^ ^]^ Polymer‐based structures have also shown the ability to fuse with living organisms,^[^ ^19^ ^]^ lipid membranes^[^ ^20^ ^]^ and form a variety of high‐density non‐lamellar phases^[^ ^21^, ^22^ ^]^ which can be utilized to mimic membrane‐rich cellular systems, including the Golgi apparatus^[^ ^23^ ^]^ and the Endoplasmic reticulum.^[^ ^24^ ^]^ Moreover, polymersomes can be engineered to respond to a wide variety of stimuli, including light,^[^ ^25^ ^]^ acoustics,^[^ ^26^ ^]^ magnetic fields,^[^ ^27^ ^]^ and temperature.^[^ ^28^, ^29^ ^]^ All of these biologically useful functionalities can be achieved by changing the composition, size, and ratio of the synthetic polymer building blocks.^[^ ^30^, ^31^ ^]^

Of the various biologically important stimuli responses that can be engineered into polymeric vesicles, temperature is particularly attractive due to its common utilization in polymer‐based drug delivery^[^ ^32^, ^33^ ^]^ and biosensing.^[^ ^34^ ^]^ The thermoresponsive properties of such systems are normally underpinned by the ability of polymers to exhibit a volume phase transition at a designated temperature defined by the polymer structure,^[^ ^35^ ^]^ which has enabled a wide range of temperature‐based functionalities. This reversible volume phase transition is caused by an alteration to the hydration state of the polymer, leading to a change in the packing factor parameter, *p*.^[^ ^36^ ^]^ Typically, most polymers exhibit a lower critical solution temperature (LCST), whereby an increase in temperature above this value results in the polymer becoming hydrophobic and thus shrinking in size.^[^ ^37^ ^]^ However, an increase in temperature above the hydrophobic block transition temperature leads to an increased chain mobility, causing a structural transformation of vesicles. This transition in hydration state and subsequent structural changes illustrate how an alteration of the *p*‐value provides a transition in the self‐assembly capabilities of the copolymer toward other morphologies.^[^ ^38^ ^]^ Such *p*‐value alteration can be used as a strategy to modify the general permeability properties^[^ ^39^ ^]^ of the polymeric assembly and membrane deformation,^[^ ^40^ ^]^ resulting in a change in the local copolymer membrane bending and curvature. Therefore, thermoresponsive polymers are an emerging class of materials in the development of synthetic cells with tailored properties and tunable functionalities.

Of particular interest is the biocompatible amphiphilic di‐block copolymer poly(ethylene oxide)–poly(butylene oxide) (PEO‐PBO) that exhibits thermally responsive properties^[^ ^41^ ^]^ and can form polymersomes.^[^ ^18^, ^42^, ^43^ ^]^ However, despite the potential of PEO‐PBO polymersomes as a delivery vector and stimuli‐responsive synthetic cell chassis, little attention has been given to the ability to utilize PEO‐PBO polymers as a component within existing synthetic cells.

**Figure 1:**
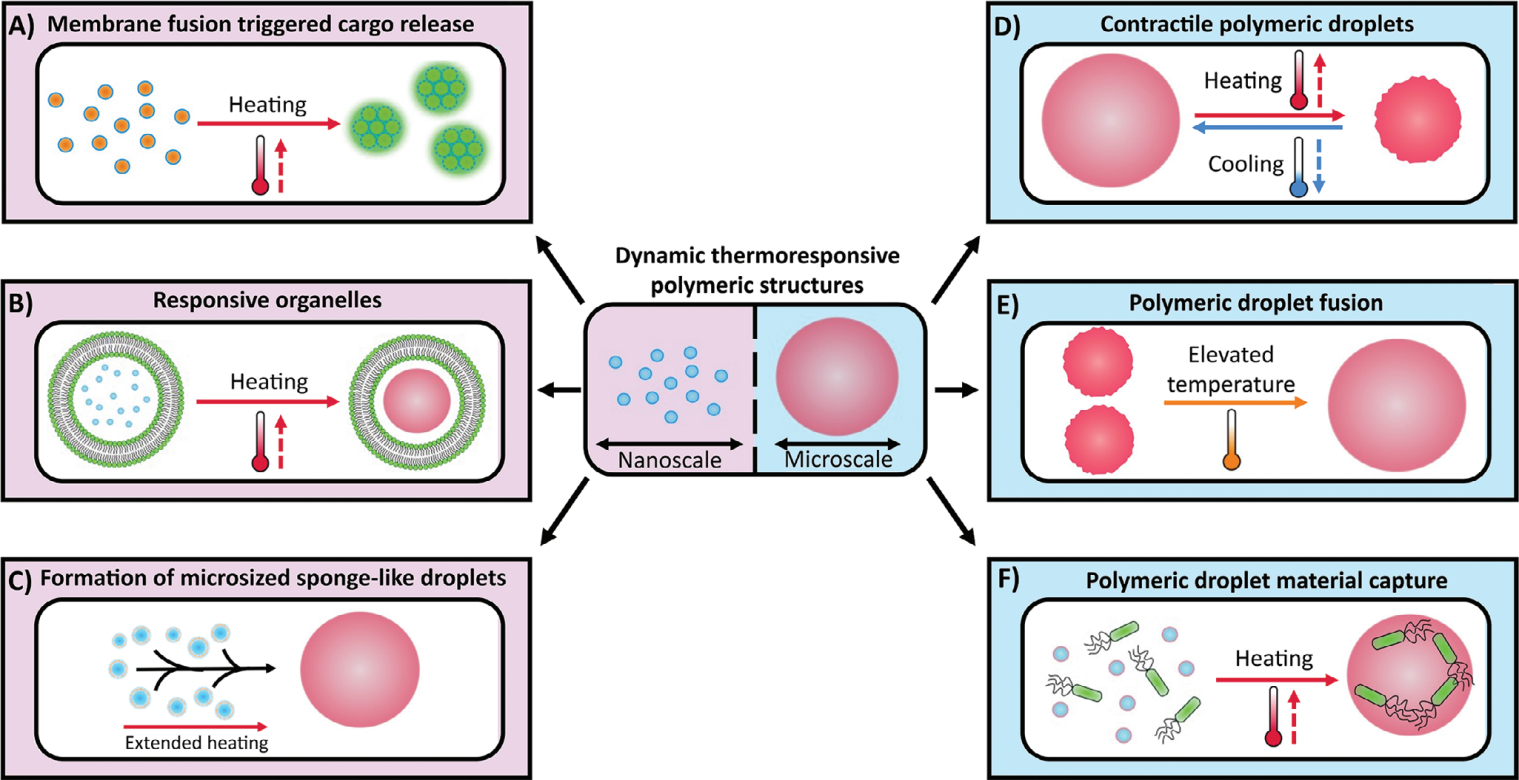
Overview of the dynamic behaviors for thermally responsive PEO‐PBO polymersomes and droplets. The dynamic behaviors are split into responses on the nanoscale (purple shading) and microscale (blue shading). On the nanoscale, the polymersomes can be utilized for cargo release (A), behave as thermosensitive organelles (B), and generate microsized sponge‐like droplets (C). Meanwhile, on the microscale, the polymeric structures exhibit thermoresponsive contractility (D), fusion between droplets (E), and material capture (F).

In this study, we introduce a novel approach by utilizing a temperature‐dependent hexagonal phase transition in PEO‐PBO polymersomes to display a range of biomimetic functionalities on both the nanoscale and microscale (**Figure** **1**), including mimicking membrane fusion, controlled cargo release, temperature‐based micron scale cellular contractility and external material capture. These functionalities are underpinned by structural changes in the polymer's arrangement promoting membrane curvature and flexibility, pivotal in the processes of cellular membrane fusion and trafficking. By mimicking this natural cellular mechanism, our system offers a novel avenue for the development of synthetic cells and organelles capable of complex interactions with biological systems, including dynamic reshaping of membranes required for intracellular transport and organization, fusion, and interaction with external objects. These findings open up new possibilities for the development of thermoresponsive polymersomes and polymeric contractile and fusogenic synthetic cells to expand the repertoire of cellular behaviors that synthetic cell structures can replicate.

## 2 Results and Discussion

### 2.1 Probing the Temperature‐Induced PEO‐PBO Structural Transition

**Figure 2:**
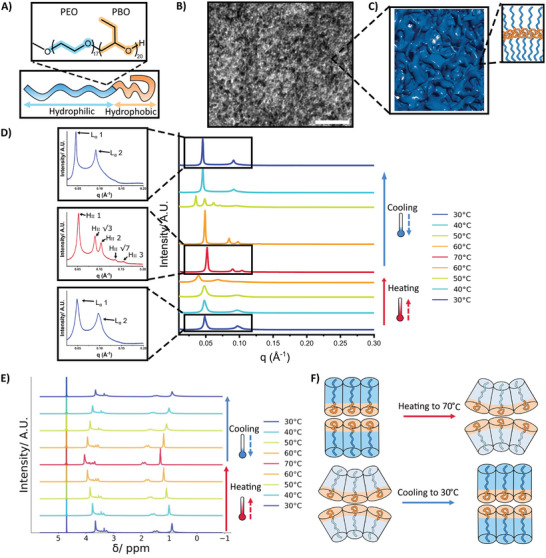
The temperature response of the PEO‐PBO polymer. A) A schematic of the chemical composition of the PEO‐PBO polymer. The hydrophobic PBO polymer is shown as the orange coiled chain of the PEO‐PBO polymer while the hydrophilic PEO polymer is shown as the blue chain. B) A Transmission electron microscopy image of the PEO‐PBO polymer, limited structural order can be seen which is consistent with sponge phases. The scale bar is 50 nm. C) A schematic showing the sponge phase of the PEO‐PBO polymer. D) A SAXS plot demonstrating how a heating/cooling cycle alters the phase behavior of the PEO‐PBO polymer. The heating and cooling portions of the graph are indicated by the thermometer schematics. Enlarged regions highlight the phases of the polymer with diagrams at the beginning, middle, and end of the cycle. Above 60 °C, an inverse hexagonal (H_II_) phase is present which disappears upon cooling. E) A ^1^H NMR plot showing how the chemical shift of the PEO‐PBO polymer alters during a heating/cooling cycle. The heating and cooling portions are indicated by the thermometer schematics in the graph. Upon heating, a shift to higher ppm is seen. Upon cooling, the signal shifts back to a lower ppm but does not fully return to the original position before the temperature cycle. F) A schematic illustrating the change in packing and curvature of the polymer during heating and cooling. Heating to 70 °C causes dehydration of the copolymer's hydrophilic block (shown by the color change), resulting in increased curvature and structural rearrangements. Cooling reverses the conformational changes, restoring the polymer's original packing and structure's curvature.

As a first experimental approach, we investigated the structural response of the PEO‐PBO polymer to temperature changes in order to elucidate the structural functionality that will be carried into our polymeric cell mimics. The PEO‐PBO copolymer self‐assembles in water into membranes, thanks to the hydrophilic poly(ethylene oxide) (PEO) brush consisting of 17 repeat units and the hydrophobic poly(butylene oxide) (PBO) segment with 20 repeat units forming the resulting membrane interior (**Figure** **2**A). Using transmission electron microscopy (TEM), we observed that at high concentrations the hydrated polymer formed a membranous network lacking long‐range order, consistent with previously observed lipid sponge phases^[^ ^44^, ^45^ ^]^ (Figure 2B,C). In this network, the PEO groups are exposed to the aqueous solution while the PBO groups are shielded from the aqueous solution by being contained within the bilayer network. Further analysis of this phase using polarising microscopy provided additional evidence of a sponge phase through the absence of birefringence (Figure S1, Supporting Information). This characteristic is indicative of the amorphous and isotropic nature of the sponge phase, corroborating our TEM findings.

The temperature‐responsive nature of the PEO‐PBO polymer was then characterized using small‐angle X‐ray scattering (SAXS) (Figure 2D). SAXS analysis of a 30 w/w% polymer dispersion revealed that the hydrated polymer initially adopts a lamellar structure (L_α_) with a d spacing of 130 Å, as indicated by the peaks with a characteristic 1: 2: 3 ratio. However, upon heating to 70 °C, the structure transitions to an inverse hexagonal (H_II_) phase, evidenced by the reflections 1: √3: 2: √7: 3 and a reduced spacing of 116 Å. The onset of this phase transition occurs at ≈60 °C. Upon cooling, the H_II_ phase disappears, reverting to the lamellar phase at the same position, though with an extended d spacing of 138 Å. The small change in d spacing upon cooling suggests a small change in the thickness of the polymer membrane, indicating an irreversible change occurs in a heating/cooling cycle as opposed to fully reversible systems which maintain their original d spacing on heating/cooling.^[^ ^46^ ^]^ The production of the H_II_ phase and its disappearance upon cooling are reversible processes linked to increased chain disorder at higher temperatures. We also observed using wide‐angle X‐ray scattering (WAXS) (Figure S2, Supporting Information) and differential scanning calorimetry (DSC) data (Figure S3, Supporting Information) that the PEO‐PBO polymer remained in a fluid state throughout the temperature cycle. This ruled out the possibility of a gel‐fluid phase transition being responsible for the thermosensitive behavior of PEO‐PBO.

The temperature sensitivity of the polymer behavior allowed us to monitor changes in the polymer chains’ self‐assembly using proton nuclear magnetic resonance (^1^H NMR) spectroscopy by observing the change in chemical shift of the copolymer's hydrogens in response to temperature variations. The resulting spectra (Figure 2E) portray the evolution of PEO‐PBO proton intensity as a function of the solution temperature, with NMR spectra collected every 10 °C during the temperature increase from 30 to 70 °C. As the temperature increases, a noticeable downfield change in chemical shift occurs due to the alteration in the polymer structure packing and increase in curvature impacting on hydrogen bonding between the polymer and complexed water molecules.^[^ ^47^ ^]^ Specifically, during the transition to the inverse hexagonal (H_II_) phase, the organization of polymer chains undergoes significant changes. In the sponge‐like phase, the polymer chains are arranged in a bilayer structure, resulting in a relatively uniform electronic environment around the nuclei. In contrast, the inverse hexagonal phase involves the formation of cylindrical structures, creating different local environments due to increased curvature and altered packing densities. This is depicted in Figure 2F. Moreover, the intensity of the polymer chains also alters upon heating (Figure S4, Supporting Information), demonstrating an alteration of the polymer solubility in water and conformational changes such as surface curvature at elevated temperatures. Upon cooling, a significant reversal in both chemical shift and intensity is observed. However, neither fully reverts back to their original values as the spectra differ between the two 30 °C recordings before and after the thermal cycle, with the intensity being lower after the cycle, again indicating some degree of irreversible structural change has also occurred (as seen with the SAXS data), suggesting a subtle transformation in the polymer environment and the formation of larger polymeric structures. These changes could be due to variations in sample concentration, such as deposition or sedimentation, or because the formation of larger structures makes the polymer chains slower to rotate.

Consequently, both the ^1^H NMR and X‐ray scattering data demonstrate the formation of a more strongly curved PEO‐PBO polymer phase with an increase in temperature. Higher temperatures result in greater chain disorder, leading to increased area occupancy for each PEO‐PBO polymer, producing curvature stress and increasing the exposure of the hydrophobic PBO segment to the aqueous environment. In order to reduce this curvature stress, more curved H_II_ phases are formed where the polymer hydrophobic section can occupy a larger area and reduce their contact with aqueous environments.^[^ ^48^ ^]^ When reducing the temperature, this curvature stress is decreased as the hydrophobic PBO segment again occupies a smaller area and the H_II_ phase becomes disfavored.

### 2.2 Temperature‐Driven Fusion of Nanoscale Polymersomes

**Figure 3:**
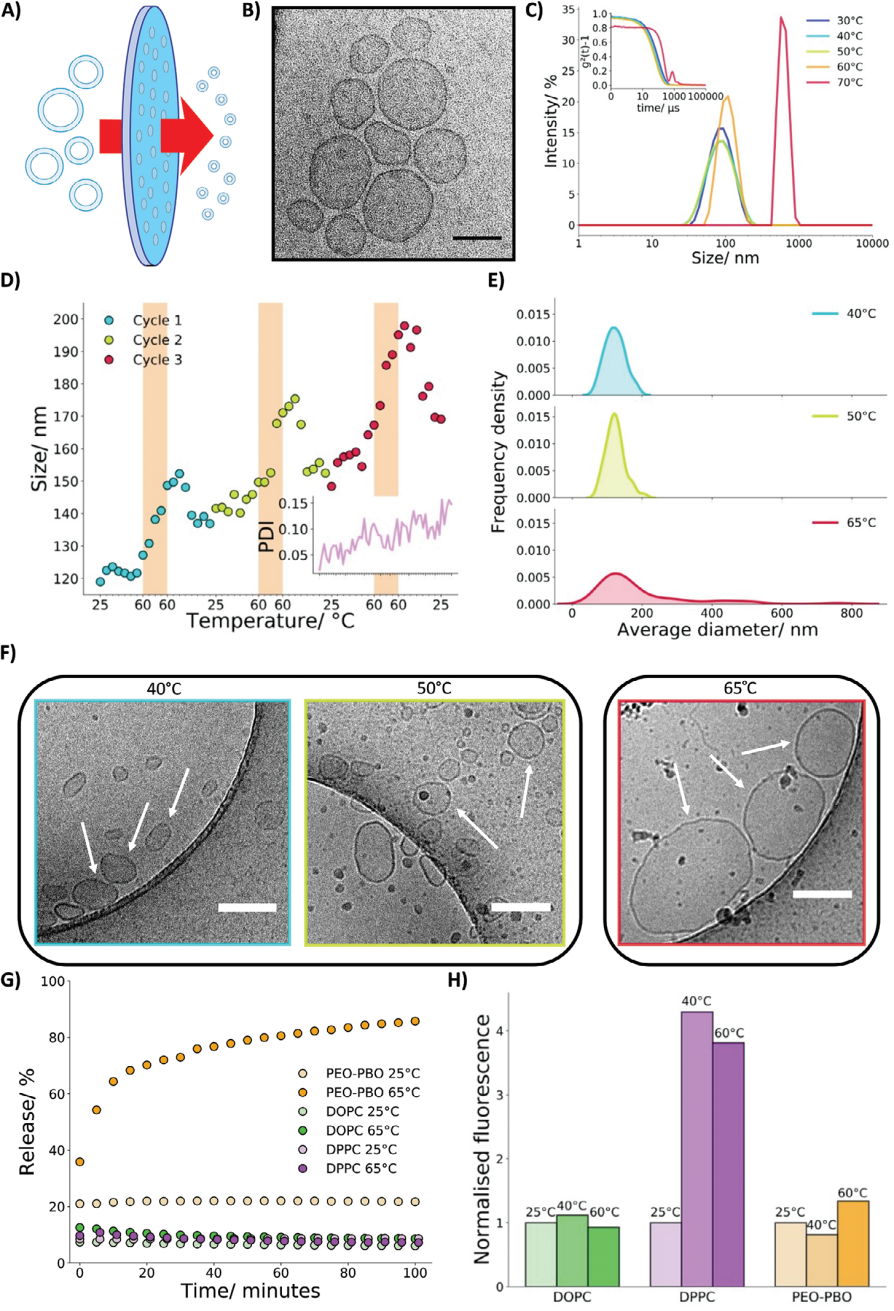
Temperature responsive behavior of PEO‐PBO polymersomes. A) Schematic showing the extrusion method for making small unilamellar vesicles (SUVs) from self‐assembled larger vesicles. B) A Cryo‐TEM micrograph of PEO‐PBO polymersomes at 25 ^°^C. The scale bar is 100 nm. C) DLS size scans with attached autocorrelation functions showing how the polymersome size changes with temperature. From 60 °C onwards, the polymersome size starts to increase. D) A size versus temperature plot illustrating the impact of heating/ cooling cycles on the size and polydispersity (PDI) of PEO‐PBO polymersomes. Repeated cycles result in an increase in both size and polydispersity attributed to polymersome fusion. The shaded region of the plot indicates where the temperature remained constant at 60 °C. E) A schematic indicating the change in size of the polymersome structures as they undergo the heating/cooling cycles. F) Representative Cryo‐TEM micrographs of PEO‐PBO polymersomes heated to 40, 50, and 65 °C. At 40 and 50 ^°^C, polymersomes were similar in size, though, at 65 °C, substantially larger polymersomes are present through the fusion of the polymersomes. The scale bars are 200 nm. G) A graph showing the release kinetics of calcein from PEO‐PBO polymersomes at 25 and 65 °C and lipid DOPC and DPPC vesicles at 25 and 65 °C. H) A bar chart comparing the temperature of calcein release from PEO‐PBO polymersomes to DOPC and DPPC vesicles. An increase in normalized fluorescence corresponds to the release of calcein from the vesicles or polymersomes. Both DPPC and PEO‐PBO show a release of calcein at their transition temperatures.

After unraveling the thermosensitive behavior of the PEO‐PBO copolymer, our next objective was to incorporate this temperature response into nanoscale polymersomes, which can be utilized as synthetic organelle mimics.^[^ ^49^ ^]^ As a result, we produced nanoscale PEO‐PBO polymersomes (**Figure** **3**A) by extrusion and investigated their responsiveness to temperature changes. Within these polymersomes, the hydrophilic PEO brush is exposed to the aqueous environment, while the hydrophobic PBO forms the interior of the polymersome membrane. The cryo‐transmission electron microscopy (cryo‐TEM) images (Figure 3B) confirmed the presence of unilamellar polymersomes exhibiting a single membrane bilayer with a thickness of ≈2.4 nm, consistent with previously reported values.^[^ ^42^ ^]^ The dynamic light scattering (DLS) analysis (Figure S5, Supporting Information) revealed an average diameter of 115 nm.

To understand the temperature‐induced behavior of the PEO‐PBO polymer in the polymersomes, we performed controlled temperature DLS experiments. We gradually increased the sample temperature from 30 to 70 °C, monitoring size changes every 10 °C (Figure 3C). No significant alterations in size were observed when samples were heated up to 50 °C. However, at 60 °C, a small increase in size occurred and at 70 °C, a clear size increase of the polymersome population was evident. This event occurred at the same temperature as the previously shown PEO‐PBO H_II_ phase transition. The corresponding autocorrelation function profiles also reflect this effect. The autocorrelation function at 70 °C had significantly shifted to larger sizes and longer decay times, further indicating an increase in polymersome size. Furthermore, incubating the polymersomes at 60 and 70 °C for 5 min (Figure S6, Supporting Information) unveiled a significant increase in size over 5 min at 70 °C compared to 60 °C, demonstrating the temperature dependency of the size increase. Upon cooling a polymersome population back down from 70 to 65, 60, and 55 °C (Figure S7, Supporting Information), the size continued to increase rather than returning to the original distribution at 60 and 50 °C, suggesting that the polymersome size increase is not fully reversible, matching observations from the ^1^H NMR analysis.

We further probed this size increase through a series of DLS experiments designed to investigate the effect of cyclic heating and cooling on polymersome size. By subjecting the polymersomes to repeated cycles of heating and cooling while staying at temperatures below the phase transition temperature (70 °C), we aimed to determine if it is possible to modulate the size increase with temperature. The polymersomes were heated from 25 to 60 °C with a 5 °C increase over 5 min and held at 60 °C for 10 min before cooling back down to 25 °C. This heating and cooling cycle was repeated for 3 cycles. The DLS experiment results (Figure 3D) revealed that while there was minimal change in the average size of the polymersomes upon heating to 60 °C, a steady increase in size was observed at this temperature over a 10‐minute time period followed by a plateau during the cooling steps above 50 °C. Even upon cooling back down to 25 °C, the average polymersome size remained larger than at the start of the cycle. The size remained almost constant over the 5‐minute period at 25 °C between cycles 1 and 2 (≈3.5% size increase) and cycles 2 and 3 (≈2.7% size increase). When compared to the size increases of 12.1%, 8.6%, and 11% observed in 6‐minute intervals at 60 °C, it is evident that the polymersomes exhibit a clear temperature dependency for size increase. This behavior highlights the importance of temperature in modulating polymersome size, demonstrating that the size increase is primarily driven by exposure to higher temperatures.

The polydispersity index (PDI) also steadily increased throughout the temperature cycling process, indicating the presence of larger structures within the polymersome solution. To further support the increase of size at relatively high temperatures (60 °C and above), particle concentration measurements for one temperature cycle were conducted (Figure S8, Supporting Information) which demonstrated a concentration decrease concurrent with an increase in size. Additionally, monitoring the solution turbidity by eye (Figure S9, Supporting Information) showed an increase upon heating. This observation was further characterized by measuring the absorbance at 400 nm during three heating/cooling cycles which revealed changes in turbidity within each cycle (Figure S10, Supporting Information), closely mirroring the DLS temperature cycling data. The decrease in turbidity during cooling can be attributed to the generation of large aggregates that deposit on the cuvette and are not detected. Overall, these findings suggest the irreversible formation of larger structures upon heating PEO‐PBO polymersomes through the polymer H_II_ phase transition, arising from fusion events. Indeed, vesicle fusion leads to the formation of larger vesicles. This process increases light scattering, resulting in a cloudier appearance and turbidity increase.

In order to unravel the morphological transformations of the larger PEO‐PBO structures formed upon heating, we performed cryo‐TEM imaging on PEO‐PBO polymersomes heated to 40, 50, and 65 °C. Subsequently, we plotted their respective size distributions for each condition (Figure 3E,F) based on micrographs capturing ≈50 polymersomes. Interestingly, the samples heated to 40 and 50 °C displayed almost identical morphologies, characterized by small single bilayer polymersomes. Additionally, their size distributions closely mirrored each other, with mean sizes of 122 and 123 nm, respectively. These observations align with the DLS distributions which showed no significant alteration in polymersome size up to 60 °C. This consistency is further supported by cryo‐TEM images obtained at 25 °C, revealing polymersomes of identical appearance. In contrast, at 65 °C (Figure 3F), the cryo‐TEM images revealed the presence of considerably larger PEO‐PBO polymersomes with a single bilayer. This resulted in a significant size distribution at larger scales (exceeding 800 nm) and an average size of 194 nm. This finding is consistent with the size increase detected in the DLS data, suggesting that the size increase arises from the fusion of smaller PEO‐PBO polymersomes into larger PEO‐PBO polymersomes, a phenomenon previously observed with PEO polymers after PEO homopolymer addition and in absence of temperature control.^[^ ^50^ ^]^

From the previously gathered X‐ray scattering and ^1^H NMR data, we can rationalize that the formation of the larger polymersomes is occurring through the generation of an energetically favorable H_II_ phase between the polymersomes at high temperatures which requires the formation of trans monolayer contacts and stalks between opposing bilayers.^[^ ^51^ ^]^ Therefore, heating the PEO‐PBO polymersome dispersion to temperatures above 60 °C produced contacts between polymersomes due to the favorability of adopting H_II_ phase mesostructures. These contacts facilitate polymersome bilayer merging and production of larger structures, as evidenced by DLS and cryo‐TEM. In particular, the DLS, through showcasing the formation of a larger structure at 60 °C before a decrease in size on cooling, suggests the formation of H_II_ phase mesostructures at elevated temperatures. In other words, the ability of the hexagonal phase to induce curvature and destabilize the membrane bilayer facilitates the dynamic reshaping of membranes by promoting and mimicking membrane bending, fission and fusion events, vital cellular processes.^[^ ^52^ ^]^ Hence, the favorability of a H_II_ phase at elevated temperatures is the predominant mechanism for PEO‐PBO membrane fusion and shows that we can program functionalities observed in bulk and which have biological relevance into our nanoscale polymersomes.

After demonstrating that the temperature‐responsive PEO‐PBO polymer can induce fusion in PEO‐PBO polymersomes, we next explored ways to exploit this functionality. Temperature is a well‐established approach of controllably releasing molecules^[^ ^53^ ^]^ from analogous lipid vesicles for drug delivery.^[^ ^54^ ^]^ Therefore, we investigated the potential of PEO‐PBO polymersomes as vectors for temperature‐dependent molecule release due to their distinct thermal response (Figure S11, Supporting Information). To demonstrate this capability, we designed experiments to monitor the release of encapsulated molecules from the polymersomes upon heating. A self‐quenching concentration of fluorescent dye calcein was encapsulated into PEO‐PBO polymersomes and they were heated to 60 °C or higher. Fluorescence dye leakage was then detected as a fluorescence increase. As the temperature increased, a notable increase in fluorescence was observed, indicating the calcein dye release into the external solution. This release was a consequence of membrane disruption during the previously described fusion events between the polymersomes as they formed a H_II_ mesostructure.

The quantification of calcein release was measured at different temperatures and the results showed that PEO‐PBO polymersomes exhibit temperature‐responsive release behavior (Figure 3G). At 25 °C, there was negligible calcein release over a 3‐hour period. However, at 65 °C, a significant release of calcein was observed, with most of the release occurring within the first 20 min and the curve plateauing at 90% after 3 h. This temperature‐responsive release behavior indicates that cargo release occurs during bilayer fusion, where the creation of stalks between bilayers produces defects through which small molecules can escape. As the release kinetics are influenced by bilayer fusion, the membrane composition or the PEO‐PBO chain lengths could alter the fusion temperature and speed, thus, enabling the facile creation of a readily tunable thermoresponsive polymersome model. The release behavior was not observed at 65 °C for 1,2‐dioleoyl‐sn‐glycero‐3‐phosphocholine (DOPC non‐thermoresponsive) and 1,2‐dipalmitoyl‐sn‐glycero‐3‐phosphocholine (DPPC thermoresponsive through a gel‐to‐fluid phase transition^[^ ^55^ ^]^) lipid vesicles, demonstrating that the polymersomes have a different release mechanism to the analyzed lipid vesicles.

In further comparison to the vesicle systems (DOPC and DPPC), the difference in the initial release percentages at 25 °C is notably significant (21% for PEO‐PBO polymersomes compared to 7% and 9% for DOPC and DPPC vesicles respectively). This observation suggests that PEO‐PBO polymersomes may carry a smaller payload than lipid vesicles due to a smaller difference between full release and no triggered release. The smaller payload could be a result of PEO‐PBO polymersomes possessing a thinner bilayer than the lipid vesicles (2.4 nm for PEO‐PBO compared to 4.5 nm^[^ ^56^ ^]^ for DOPC and 4.1 nm^[^ ^57^ ^]^ for DPPC), facilitating the more accessible translocation of cargo across the bilayer. However, the thinner bilayer of PEO‐PBO may also enable a large release upon polymersome fusion. This is due to the requirement for only a small defect to be present within the membrane to connect the external solution to the polymersome lumen, facilitating a more efficient release mechanism.

To comprehensively assess the thermosensitive release properties of PEO‐PBO polymersomes in comparison to common lipid vesicle compositions, a temperature gradient experiment was also conducted (Figure 3H; Figure S12, Supporting Information). It was observed that the non‐thermosensitive DOPC vesicle did not exhibit any cargo release as expected, while the thermoresponsive DPPC vesicle released a substantial payload at ≈40 °C, corresponding to the gel‐to‐fluid transition temperature of DPPC.^[^ ^55^ ^]^ In contrast, the PEO‐PBO polymersomes demonstrated cargo release only at 60 °C, above the observed PEO‐PBO phase transition where a significant change in fluorescence was observed. Furthermore, the magnitude of the PEO‐PBO release event was much smaller than that of DPPC vesicles, further confirming a smaller payload retained within the PEO‐PBO polymersomes compared to lipid vesicles. These dye‐leakage results demonstrate the prompt stimuli responsiveness of the system to thermal changes and confirm the temperature transition point observed in our previous data. Furthermore, the results show our PEO‐PBO polymersomes exhibit a different method of release (membrane fusion) in comparison to other thermosensitive lipid vesicles (gel‐to‐fluid transitions and defect formation^[^ ^55^ ^]^) and polymersomes (membrane pore formation^[^ ^28^ ^]^).

### 2.3 Fusogenic Polymersomes as a Tool for Synthetic Cell Development

Having demonstrated the thermally induced functionality of PEO‐PBO polymersomes on the nanoscale, we aimed to leverage this fusogenic property on larger length scales to create structures that can better mimic cellular form and functionality. Micro‐sized lipid‐based synthetic cells have been utilized in advanced applications such as triggering new blood vessel formation^[^ ^58^ ^]^ and as monitors for fluoride in water samples.^[^ ^11^ ^]^ However, lipid synthetic cells require formulations containing a range of set biological components to replicate dynamic sophisticated processes, limiting their versatility. In contrast, polymeric structures, such as PEO‐PBO polymersomes, and other amphiphilic structures such as dendrimers^[^ ^59^ ^]^ and other co‐polymers,^[^ ^60^ ^]^ can recreate these processes using customizable formulations, often containing fewer components. This advantage allows for greater flexibility and adaptability in designing systems for specific applications. Employing PEO‐PBO polymeric structures at a larger length scale has the potential to expand the range of applications in which synthetic cells can be used and finely customize the system's properties through the ability to readily alter the PEO‐PBO polymer.

**Figure 4:**
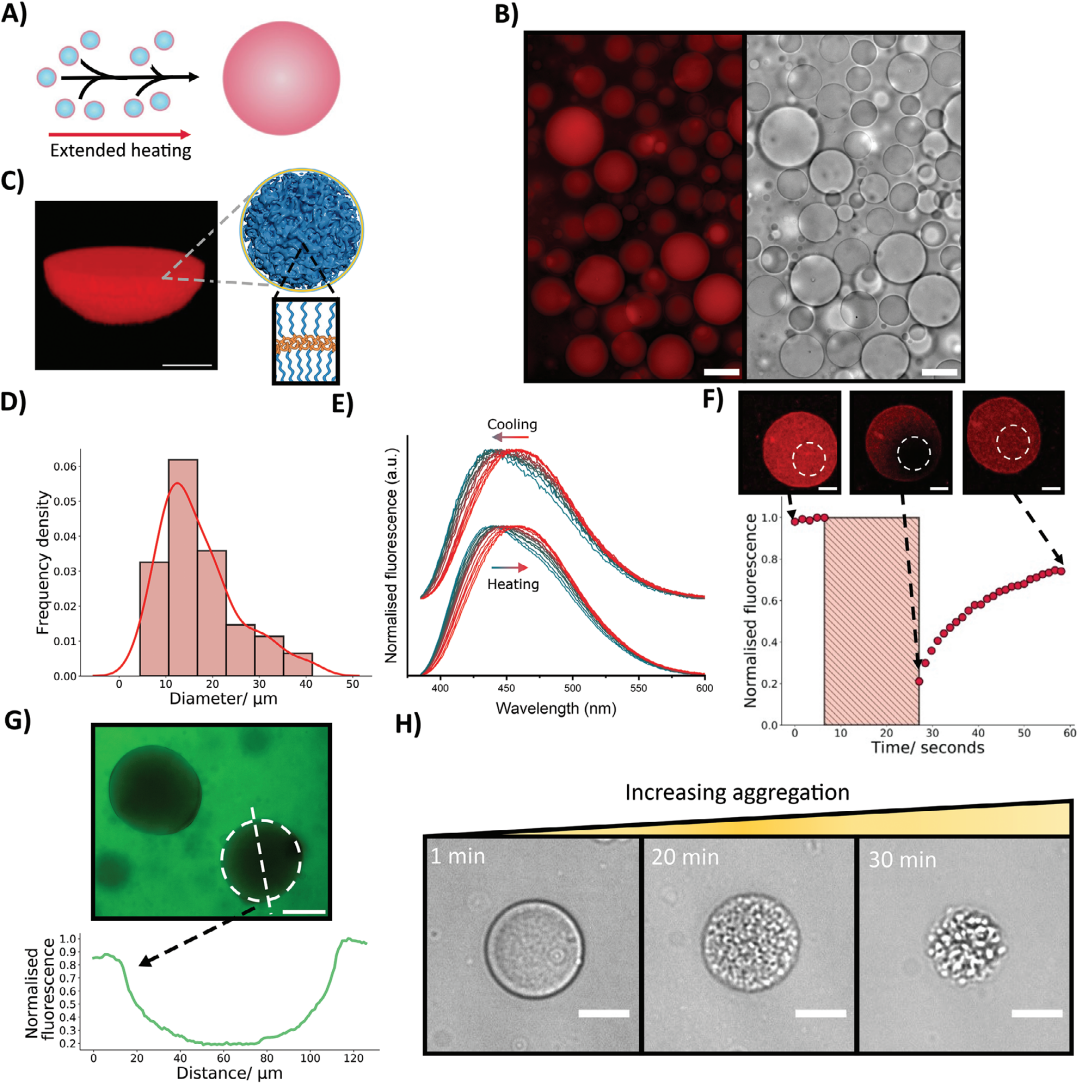
Utilizing fusogenic PEO‐PBO polymersomes for synthetic cell construction. A) A schematic illustrating that through using a temperature ramp of 5 °C a minute to 70 °C, a 5‐minute incubation at 70 °C, and cooling back to room temperature the PEO‐PBO polymersomes assemble into larger polymeric droplet structures. B) Fluorescence and brightfield images of a population of spherical polymeric droplets stained with Nile Red. The fluorescence is localized to the droplets. The scale bars are 25 µm. C) A confocal fluorescence 3D Z‐stack showing half of a polymeric droplet. The fluorescence signal originates from a lipophilic rhodamine dye incorporated within the PEO‐PBO bilayer. The accompanying schematic highlights the spongelike phase and polymer bilayer structure of the polymeric droplets. Scale bar is 10 µm. D) A histogram with a Kernal density estimation fit showing the mean diameter of a population of polymeric droplets was 17 µm with a polydispersity index of 0.24. n = 100 polymeric droplets were measured. E) Laurdan assay (excitation = 360 nm) reveals a red shift in the fluorescence spectrum upon heating, indicating Laurdan gains easier access to water molecules at elevated temperatures. Upon cooling back to 25 °C, the spectrum shifts blue again, suggesting the reformation of a membrane‐based structure in the droplets during the process. F) A fluorescence recovery after photobleaching graph with embedded confocal microscopy images showing the recovery of fluorescence within a polymeric droplet, indicating the droplet is comprised of a fluid polymeric membrane structure. The scale bars are 10 µm. G) Calcein permeation of polymeric droplets. A fluorescence image and corresponding line profile taken 60 min after the addition of 0.5 µm calcein to the polymeric droplet solution shows darker regions in the fluorescence corresponding to the positions of the droplets. These dark areas indicate limited calcein permeation into the sponge droplet interior, demonstrating that the polymeric membrane effectively separates the internal and external aqueous solutions successfully. The dashed line shows the line profile taken for the plot The scale bar is 50 µm. H) Brightfield images demonstrating a formation timelapse of larger PEO‐PBO aggregates within lipid vesicles upon heating. The scale bars are 25 µm.

To generate micron‐scale polymeric structures using PEO‐PBO polymersomes which could be employed as synthetic cells, the nanoscale vesicles underwent a controlled temperature ramp. The temperature was increased at a rate of 5 °C per minute until reaching 70 °C. Subsequently, a 5‐minute incubation at 70 °C was followed by rapid cooling to room temperature. This thermal treatment was designed to induce the fusion events between the nanoscale structures and formation of microscale polymeric structures which could be utilized as synthetic cell mimics (**Figure** **4**A). Lower temperature ramps led to no formation of polymer droplets as the polymersomes remained stable below the polymer transition temperature (Figures S13 and S14, Supporting Information).

To confirm the origin of the micron scale polymeric structures from the nanoscale polymersomes, the lipophilic rhodamine B^[^ ^61^ ^]^ and Nile red dyes^[^ ^62^ ^]^ (Figure S15, Supporting Information) were loaded within the PEO‐PBO polymersomes’ membrane. Fluorescence and brightfield microscopy imaging (Figure 4B; Figure S16, Supporting Information) revealed the appearance of clear vesicle‐like structures displaying a localized fluorescent signal. It needs to be noted that the micrometer polymeric structures were derived solely from the nanoscale PEO‐PBO polymersomes precursors containing the lipophilic dyes within the polymersome bilayer, as no other vesicles were present in the initial solution. Notably, the confocal imaging in Figure 4C demonstrated an even florescent signal throughout the microscale polymer structures, lacking the localization of signal to a single membrane that might be expected in analogous lipid vesicles used as synthetic cells,^[^ ^63^ ^]^ potentially indicating that the micron scale polymeric structures were spongelike polymeric droplets, matching the sponge phase observed in PEO‐PBO polymers in bulk. The micron‐scale polymeric structures had a broader size distribution (Figure 4D) and greater polydispersity index (0.24) than the nanoscale polymersomes. We attribute this to the many fusion events occurring between polymersomes of different sizes^[^ ^64^ ^]^ used to generate the polymeric droplets. After each fusion event, a larger size distribution would be expected.

To confirm the structure of the polymeric droplets, we performed two distinct experiments: a Laurdan assay and fluorescence recovery after photobleaching (FRAP) experiment. The Laurdan assay of polymeric SUVs (Figure 4E) showed a small and reversible spectral shift during a heating‐cooling cycle. At a higher temperature, the Laurdan dye could access water and undergo dipolar relaxation more readily.^[^ ^65^, ^66^ ^]^ This trend was the same as observed for the Laurdan dye in a bulk solution. (Figure S17, Supporting Information), showing that there is a limited bilayer structural change within the SUVs upon heating and cooling, indicating structural consistency throughout the process. This also confirmed that the thermal cycle was fully reversible, with both pre‐ and post‐cycle structures remaining bilayers^[^ ^45^ ^]^ in agreement with the SAXS data. The spectral shift of polymer was also different to a range of thermoresponsive and non‐thermoresponsive lipids (Figure S18, Supporting Information), highlighting the difference in thermoresponsive behavior and structural dynamics between the polymer and lipids.

Meanwhile, the FRAP experiments were performed on polymeric droplets stained with the lipophilic dye Nile red^[^ ^62^ ^]^ (Figure 4F) (Video. S1, Supporting Information). The analysis showed a quick recovery of membrane fluorescence, indicating that the droplets comprised of a single polymeric membrane network, instead of a collection of aggregated polymeric vesicles where limited fluorescence recovery would be observed. To verify that the polymeric droplets were comprised of a single polymeric membrane formed from the fusion of nanoscale polymersomes, we incubated two populations of polymersomes together at 70 °C, one population contained a Rhodamine dye and the other contained a Cy5 dye. The formed polymeric droplets (Figure S19, Supporting Information) could be seen to possess both fluorescent signals which were both evenly spread across the droplets, showing that both polymersome populations were present in the final droplets and the membranes of the different populations had fused together. These experiments further indicate that the polymeric droplets have a polymer‐dense, spongelike interior and thus mimic some properties of membrane‐rich cellular structures, for instance, the endoplasmic reticulum.^[^ ^24^ ^]^ To further confirm the membrane‐rich structure of our droplets, we tested their permeability to calcein, a hydrophilic fluorescent dye commonly used as a membrane integrity probe. The polymeric sponge‐like droplets were impermeable to the small molecule dye calcein (Figure 4G; Figure S20, Supporting Information), indicating the presence of intact membranes separating the polymer droplet interior from the external solution. Its exclusion from the droplet interior strongly suggests that the membrane surrounding the polymer droplets is continuous and the sponge structure acts as a robust barrier. The demonstrated dynamic production of microscale polymeric droplets exclusively in aqueous media, avoiding membrane contaminants such as oils,^[^ ^67^ ^]^ opens avenues for the potential reconstitution of membrane proteins^[^ ^68^ ^]^ into contaminant‐free polymeric bilayers on the microscale. This capability is a vital tool for constructing complex and accurate synthetic cell mimics.

To expand the potential applications of fusogenic PEO‐PBO polymersomes into more complex micron‐scale systems, a compartmentalized architecture was engineered by encapsulating nanoscale PEO‐PBO polymersomes within a DOPC giant vesicle system (Figure 4H). This approach enabled us to demonstrate and observe the fusiogenic capabilities of our polymersomes within a compartmentalized system, providing a method to template the formation of our sponge‐like microstructures within a microscale compartment. This dynamic compartmentalized system allowed the polymersomes to function as organelles within the synthetic cell analog. To elicit a response from the organelles, the sample was heated to 60 °C, a temperature where the polymersomes fuse and release cargo if desired. As a consequence, it was shown that aggregation gradually occurred over time within the lumen of the DOPC vesicle, resulting in the formation of a PEO‐PBO sponge droplet whose size increased over time. While further investigation needs to be done to elucidate the crowed environment and concentration effect on the fusion within the lipid membrane, this work demonstrates that PEO‐PBO polymersome organelles can be thermally triggered to interact synergistically with existing compartmentalized synthetic cell structures. This paves the way for the development of more complex polymersome‐lipid hybrid soft matter systems.

### 2.4 Exhibiting Contractility within Polymeric Synthetic Cell Analogues

**Figure 5:**
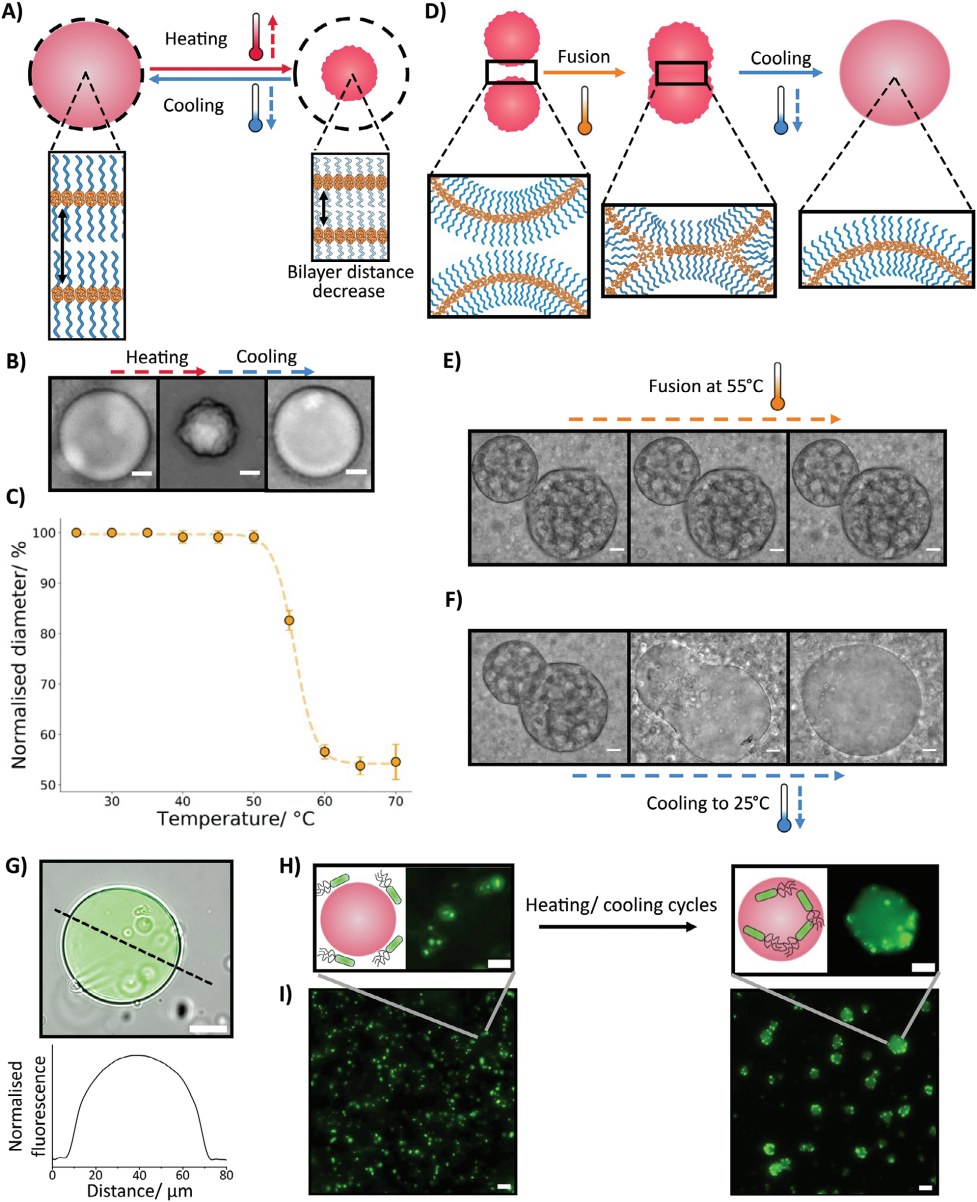
Functionality and applications of contractile PEO‐PBO polymeric droplets. A) An illustration indicating the reversible contractility observed within PEO‐PBO polymeric droplets. The dotted circle shows the relative size of the polymer droplet before and after the contraction. The schematic also highlights the reversible decrease in bilayer distance upon heating due to the expulsion of water, leading to a size decrease. B) Brightfield images showing the contractility of PEO‐PBO polymer droplets, upon heating to 70 °C the droplets significantly shrink, this is reversed upon cooling back to 25 °C. The scale bar is 10 µm. C) A plot demonstrating how the size of PEO‐PBO polymeric droplets alters with temperature, a significant shrinkage is seen between 50 and 60 °C. The error bars were obtained from the standard deviation of *n* = 3 PEO‐PBO polymeric droplets. D) Schematic illustrating the fusion of semi‐contracted PEO‐PBO polymeric droplets at an elevated temperature before the formation of a larger uncontracted droplet upon cooling. During the fusion event, the different polymeric bilayers fuse together. E) Brightfield images showing the timelapse of a fusion event between two semi‐contracted PEO‐PBO polymeric droplets at 55 °C. The scale bar is 20 µm. F) Brightfield images presenting the formation of a singular PEO‐PBO polymeric droplet created from the two fused semi‐contracted polymeric droplets upon cooling. The scale bar is 20 µm. G) An overlapped fluorescence and brightfield microscope image showing the successful encapsulation of calcein. The green color indicates the calcein channel while the plot shows the relative calcein fluorescence change along the black dashed line. Scale bar is 20 µm. H) Drawings with accompanying fluorescence images showing how PEO‐PBO polymersomes can interact with bacteria, with multiple heating/cooling cycles the bacteria are taken up into the produced polymer droplet structure. The scale bars are 10 µm. I) Fluorescence population images of GFP‐expressing bacteria before and after trapping in polymeric droplets. Prior to the heating and cooling cycles, the bacteria are present throughout the sample, while after, the bacteria are localized within the produced polymer droplets. The scale bars are 20 µm.

To further harness the potential of PEO‐PBO polymersomes in creating complex synthetic cell systems, we explored additional functionalities beyond their fusogenic capabilities. In addition to their fusogenic ability, microscale PEO‐PBO polymer droplets exhibit contractility, another crucial feature present in biological systems^[^ ^69^ ^]^ that can be mimicked using simple polymer synthetic cells. Here, we demonstrated contractility by gradually heating the polymeric droplet sample to 70 °C at a rate of 1 °C per minute, as shown in **Figure** **5**A–C and Videos S2,S3 (Supporting Information). Remarkably, the PEO‐PBO polymeric droplet retains its structure upon heating to 50 °C, consistent with the behavior observed in smaller nanoscale polymersomes. However, as the sample approached the polymersome transition temperature ≈60 °C, a substantial size contraction of ≈44% is observed, resulting in the PEO‐PBO polymeric droplet possessing a crumpled appearance with a darker texture. The crumpled individual droplets remained stable over a period of minutes whilst the experiments were performed. This distinct change in optical texture again supports the argument that solid polymeric droplets with a membrane rich interior are being produced at higher temperatures instead of more transparent single‐membrane polymeric synthetic cells. Subsequently, heating the sample above 60 °C does not further reduce the size. Remarkably, upon cooling back to 25 °C, the polymeric droplet returns to its original shape and appearance, demonstrating that this transition is fully reversible. Further characterization revealed that the contractility occurred on a timescale of seconds (Figure S21, Supporting Information). These results confirm that the polymeric droplets exhibit thermosensitive properties within the same temperature range as the smaller polymersomes, further demonstrating that the polymeric droplets are formed from the fusion of PEO‐PBO polymersomes. At this length scale, the thermoresponsive behavior is analogous to other polymeric systems such as poly(N‐isopropylacrylamide) (PNIPAM) microgels^[^ ^70^ ^]^ and arises from the increased hydrophobicity of polymer bilayer at this temperature^[^ ^71^ ^]^ which leads to the expulsion of water from around the polymer and the favoring of polymer‐polymer interactions, causing the crumpling of the polymeric droplets. The development of these contractile polymeric droplets paves the way for their potential usage as synthetic cell analogs to mimic contractile biological entities, for instance, cardiomyocytes,^[^ ^72^ ^]^ which contract multiple times per minute,^[^ ^73^ ^]^ and which are challenging to mimic using conventional lipid synthetic cells.

To showcase the potential applications of PEO‐PBO polymer droplets in their utilization as synthetic cells, we investigated their contractility and demonstrated their ability to fuse together and to trap bacteria. Combined, this provides evidence that the PEO‐PBO polymeric droplets can function as complex soft matter systems by themselves or interact with biological entities in a constructive manner, which is fundamental to construct increasingly sophisticated synthetic cell mimics and expanding the applications of synthetic cell technologies.

To show that controlled polymeric droplet fusion can be readily achieved in the PEO‐PBO polymeric droplets, we fused together two partially contracted polymeric droplets by heating the sample to 55 °C, a temperature where the polymeric droplets are partially shrunk and possessed hydrophobic character (Figure 5D). When two partially shrunken polymeric droplets made contact at this temperature, their hydrophobic profile increased due to the exposure of hydrophobic parts and dehydration of hydrophilic brushes. This enhanced hydrophobic interaction facilitates the droplets coming into close proximity, followed by fission and eventually leading to their fusion and gradual merging (Video S4, Supporting Information; Figure 5E). This was comparable to the increase in size phenomena showcased by the nanoscale polymersomes aggregating at elevated temperatures due to the favorability of a H_II_ phase. Merging events were only observed when the polymeric droplets were partially shrunken, showing the requirement for the droplets to possess some hydrophobic character, and demonstrating how temperature can modulate the fusogenic and overall dynamic behaviors. To confirm bilayer fusion, we cooled the sample back down to 25 °C resulting in the reswelling of the polymeric droplets and the production of one large droplet instead of two smaller ones (Figure 5F; Video S5, Supporting Information). This again replicated the increase in polymersome size on the nanoscale obtained through a heating/cooling cycle showing that the PEO‐PBO droplets have a behavior analogous to that of observed at the nanoscale, enabling the easy extrapolation of complex nanoscale functionality into microscale synthetic cell structures.

It is important to note that the contractile behavior is observed at a higher temperature (70 °C) and is not present at 50 °C. The fusion event requires the polymersomes to possess a partial hydrophobic character which only occurs when the polymersomes are in a partially shrunken state at a lower temperature (≈50 °C). Therefore, when at this temperature, two partially shrunken polymersomes are in close proximity to each other they will enter into contact and begin to fuse with each other in order to reduce the polymersome total surface area that is exposed to the surrounding aqueous environment. This means that the shrinking/ swelling behavior can be isolated from the fusion process by controlling the proximity of the polymersomes to each other, but the fusion process is dependent on the shrinking of polymersomes first and hence cannot be decoupled from the shrinking/ swelling and temperature control. Therefore, through modulating the temperature, the balance between the degree of shrinking and fusogenicity can be controlled. This ability to fine‐tune the temperature allows us to selectively induce either fusion or contractility, depending on the desired application.

An intriguing phenomenon observed during phase transition and droplet formation, is the ability of the droplets to trap external objects, such as biological cells dispersed in the environment, demonstrating dynamic interactions with their surroundings. The ability of PEO‐PBO polymer droplets to interface with different materials, from small molecules to single cells, was further explored (Figure 5G–I). In Figure 5G, encapsulated calcein is visible inside the sponge droplet. This calcein entrapment was obtained by mixing calcein and SUVs prior the droplet formation and after tuning the temperature to induce droplet formation, the calcein signal remained clearly detectable inside the droplet, confirming successful calcein encapsulation. Additionally, we placed nanoscale PEO‐PBO polymersomes in a solution containing *E. coli* bacteria expressing GFP to further demonstrate the droplets’ capacity to interact with larger biological entities. At 25 °C the bacteria were dispersed throughout the solution and did not interact with the PEO‐PBO polymersomes. However, when the sample underwent heating/cooling cycles, the bacteria were entrapped within the PEO‐PBO polymer droplet structure. This is evident from the localization of bacteria in Figure 5I to the PEO‐PBO droplets. To further highlight the ability of the polymeric droplets to trap objects, we formed polymeric droplets with the same droplet architecture shown previously in more physiologically relevant LB media (Figure S22, Supporting Information). The bacteria could also be localized with these droplets, demonstrating the polymer droplets had similar properties in these conditions and that bacteria can be trapped in the presence of other proteins that could absorb to the polymer membrane (Figure S23, Supporting Information). The entrapment of bacteria occurred because bacteria tend to adhere to moderately hydrophobic substances,^[^ ^74^ ^]^ which the PEO‐PBO polymersomes possess at the employed temperatures. The rupture of the bacteria cell wall and membrane structure alteration at elevated temperatures^[^ ^75^ ^]^ may also aid in the adhesion process. Consequently, when the PEO‐PBO polymersomes began to fuse at 70 °C, the adhered bacteria were also fused into the microscale polymer droplets and stayed trapped within the polymeric structure after the cooling process. The results demonstrate a proof‐of‐concept experiment aimed at showcasing the trapping capability of the system when exposed to a biological sample and provide an alternative mechanism for trapping bacteria in synthetic cell mimics.^[^ ^76^, ^77^, ^78^ ^]^ Our approach highlights the trapping mechanism's versatility, as the entrapped bacteria's viability does not limit the potential applications. Dead or inactivated bacteria can still provide functional surfaces for biohybrid applications. Additionally, the trapping mechanism is advantageous for rapidly removing bacteria from solutions in applications such as sterilization or environmental cleanup, regardless of bacterial viability. Currently, the employed temperature used to cause bacteria entrapment (70 °C) will lead to the inactivation of many bacterial species.^[^ ^79^ ^]^ However thermophilic bacteria, which can produce a range of thermostable enzymes for uses in food processing and paper bleaching for instance,^[^ ^80^ ^]^ would be able to function upon entrapment. Consequently, PEO‐PBO polymeric droplets can be used as synthetic cell analogs to interface with biological structures in a controlled and stimuli‐driven manner, which is useful in creating novel hybrid systems.^[^ ^81^ ^]^ Moreover, the controlled adhesion of bacteria to PEO‐PBO polymer droplets could be advantageous as a method of cleaning and removing bacteria from solutions. Future experiments will focus on optimizing the temperature conditions to broaden the range of bacteria species that can be entrapped without inactivation. To this end, experiments are currently ongoing to decrease the system's transition temperature. This will allow to explore the use of these polymeric droplets in a broader range of applications such as drug delivery, biosensing, and environmental remediation, effectively creating a versatile synthetic cell/biohybrid technology that can seamlessly integrate with biological systems.

## 3 Conclusion

In this study, we have demonstrated a range of versatile and dynamic behaviors in PEO‐PBO polymersomes and polymer droplets by showcasing that a variety of biomimetic behaviors can be present within one system, highlighting their potential as synthetic cell analogs. By leveraging the fusogenic properties inherent in the PEO‐PBO polymer, we engineered nanoscale polymersomes capable of controlled cargo release and formation of polymeric droplets that can function as synthetic cell analogs. Furthermore, we have illustrated that these droplets exhibit contractility and can effectively trap bacteria, indicating their ability to interact dynamically with biological systems. The ability of these polymer systems to interface with bacteria opens new avenues for creating biohybrids with dynamic and responsive characteristics, thereby expanding the potential systems that PEO‐PBO polymeric structures can mimic. We believe that the exploration and manipulation of such dynamic behavior across different length scales will enable the incorporation of thermosensitive structures in a variety of ways, including small nanoscale compartments in cellular structures and cellular elements within tissue‐like entities, all crucial platforms for replicating complex living systems. Future avenues include reconstituting membrane proteins into polymeric droplets using protein‐containing PEO‐PBO polymersomes to increase the biomimicry of the system and altering the system's membrane composition in order to reduce the temperature at which the thermally driven events occur and increase the therapeutic and biological relevance of the structures which is currently limited due to the high temperature of activation. However, it is worth noting that certain biological elements, such as thermophilic enzymes, extremophiles, and some heat‐stable proteins, can survive and function at higher temperatures. These elements can potentially be incorporated into our system, allowing it to be utilized in specific high‐temperature applications where these biologically robust components are beneficial. Overall, these findings underscore the potential for advancing complex synthetic cell development and the biotechnological applications that can emerge from the exploitation of the thermosensitive dynamic behavior present in PEO‐PBO polymersomes and polymeric droplets.

## 4 Experimental Section

### Materials

All materials were obtained from Sigma‐Aldrich (Gillingham, UK) and used as received unless otherwise specified.

### Transmission Electron Microscopy

A 2% uranyl acetate (UA) solution was employed as a negative staining agent. Five microliters of polymer dispersion were applied to glow discharged 300 square mesh copper grids (Agar Scientific). After 1 minute, the grids were blotted with filter paper and immersed in the UA staining solution for 20 s. Subsequently, the grids were blotted again. Imaging was performed using a JEOL LEM‐2100F equipment with a Gatan Orius SC 1000 camera (2 × 4k).

### Polarizing Microscopy

A Nikon Eclipse E600 polarization microscope with a Ximea MQ022CG‐CM camera and a Linkam LTS 350 heating stage were used for the polarizing microscopy experiments. The copolymer mixture was hydrated to 30 wt.% and sandwiched between glass slides for imaging. The samples were then imaged at 25 °C.

### X‐Ray Sample Preparation and Measurements

A 30 w/w% PEO‐PBO copolymer dispersion in PBS was placed into 1.8 mm inner diameter glass capillaries that were sealed with epoxy. The sealed capillaries were heat cycled to ensure homogenous mixing while minimizing water loss. Small‐angle X‐ray source was from B21 at Diamond Light Source with a wavelength of 0.9464 Å, energy at 13.1 keV and the samples are 3688.3 mm to the detector. While wide‐angle X‐ray diffraction data were obtained for the mixed samples using beamline I22 at Diamond Light Source. Diffraction patterns were collected using an X‐ray energy of 12 keV (wavelength 1Å) and a sample‐to‐detector distance of 162 mm for WAXS. The raw data was corrected and processed using the procedure developed by the beamline technicians at Diamond.^[^ ^82^ ^]^

### Differential Scanning Calorimetry

DSC was performed using a Perkin Elmer Pyris Diamond DSC. A known weight of 70 wt.% hydrated PEO‐PBO polymer was placed in a hermetically sealed aluminum DSC pan purchased from PerkinElmer. Before being placed in the pan the sample was subjected to 5 mixing vortexes to ensure complete content mixing. Samples were warmed from room temperature to 60 °C and held for 2 min, then cooled to 20 °C and held for 2 min before warming back to 60 °C, then held for 2 min. The entire protocol was repeated at least once to ensure reproducibility. Temperatures were scanned at a rate of 1 °C min^−1^.

### 1H NMR

^1^H‐NMR was used to monitor the intensity evolution of PEO and PBO protons peaks as a function of temperature by using a Bruker 400 MHz instrument. Block copolymer solutions were prepared in deuterated water using the same protocol described below for all the PEO‐PBO dispersions. The NMR spectra were recorded at 10 °C intervals as the temperature increased from 30 to 70 °C and then cooled back to 30 °C.

### Production of PEO‐PBO Polymersomes

PEO‐PBO polymersomes were produced by dissolving the diblock copolymer with 17 PEO repeat units and 20 PBO repeat units at a concentration 5 mg mL^−1^ in chloroform. From this solution, a film was made through evaporation of the solvent under a stream of N_2_. The films were then left under vacuum. After re‐hydrating the polymeric film with PBS at a concentration of 5 mg mL^−1^, freeze‐thaw cycles were performed five times to obtain a unilamellar dispersion of the polymersomes, this involved flash freezing the sample with liquid N_2_ before thawing with a heat gun and vortexing for 60 s at room temperature. The resulting dispersion was then extruded 21 times through a 0.1 µm polycarbonate membrane to produce PEO‐PBO polymersomes with a diameter of ≈115 nm.

To incorporate a lipophilic dye into the polymersomes’ bilayer, 0.1 mol% of octadecyl rhodamine B, 0.6 mol% of Nile Red or 0.1 mol% of Cy5.5 were added to the polymer solution in chloroform before film production, and subsequent hydration.

### Cryogenic Transmission Electron Microscopy

The samples were vitrified using a Vitrobot Mark IV (FEI) system, with precise control over temperature (21 °C) and humidity (100%). A 4 µl sample was deposited onto Quantifoil copper grids with 2 µm holey‐carbon on 200 square mesh (Agar Scientific) and then vitrified by immersing the grid in liquid ethane and subsequently transferring it to liquid nitrogen. The grid was then rapidly placed in a cryogenic stage and maintained at −180 °C. Micrographs were obtained using a Gatan 626 cryogenic holder on a FEI Tecnai 12 twin TEM operating at 120 kV with a TVIPS F216 CCD camera.

### Dynamic Light Scattering

A Malvern Zetasizer Ultra instrument (Malvern, UK) with a 632.8 nm HeNe gas monochromatic laser was used to analyze the samples. The copolymer and lipid dispersions were analyzed at a concentration of 0.1 mg mL^−1^ using a polystyrene dispensable cuvette (Malvern Panalytical, DTS0012). The scattered light was measured at an angle of 173°. All the size and concentration data were collected using multi‐angle DLS (MADLS) analysis. The particle concentration measurement process is fully automated and obtained from the Malvern Panalytical Zetasizer Ultra. All the DLS data were processed using Dispersion Technology Software (Malvern Panalytical) and the size distribution were shown considering the intensity‐weighted particle size distribution.

Heating‐cooling cycles were performed to study the size and polydispersity index (PDI) of the PEO‐PBO polymersomes using dynamic light scattering (DLS). The DLS was programmed to record the size and PDI every 5 °C, with an equilibration time of 300 s, starting from 25 to 60 °C. Subsequently, five recordings were performed at 60 °C, with a waiting time of 120 s. Finally, the DLS was programmed to record the size and PDI every 5 °C, with an equilibration time of 300 s, from 60 to 25 °C.

### Turbidity Measurements

To monitor the solution turbidity, the absorbance at 400 nm was measured using a UV‐vis spectrophotometer (Cary 3500) during 3 heating‐cooling cycles. The temperature was controlled using a programmable temperature controller and the measurements were performed in a quartz cuvette. The measurement was initiated at 25 °C and then the temperature was raised to 70 °C with the recording of absorbance at every 5 °C interval. The measurement was then reversed from 70 to 25 °C with the same interval. This process was repeated for three cycles. PEO‐PBO polymersome dispersion was prepared in PBS at room temperature with a concentration of 5 mg mL^−1^.

### Production of DOPC, POPC and DPPC Lipid Vesicles

1‐palmitoyl‐2‐oleoyl‐glycero‐3‐phosphocholine (POPC), DOPC, and DPPC lipid vesicles were created by dissolving appropriate amounts of lipid in chloroform. This was typically 5 mg for both lipid compositions. The chloroform was then evaporated under a gentle stream of N_2_ to produce a film. The resultant films were then left under vacuum overnight to remove the residual chloroform. The films were then rehydrated to the concentration of 5 mg mL^−1^ with PBS buffer. 5 cycles of freeze‐thawing were then used to produce unilamellar lipid vesicles; this was done through flash freezing the sample with liquid N_2_ before thawing with a heat gun and vortexing for 60 s at room temperature. The resulting vesicles were then extruded 21 times through a 0.1 µm polycarbonate membrane to produce POPC, DOPC and DPPC vesicles of ≈100 nm.

For the calcein encapsulation studies, 50 mm of calcein dye was also added to the film hydration solution. After production of the calcein‐containing vesicles, the vesicles were passed through a size exclusion column to remove the unencapsulated calcein. This involved using a Sephadex G‐50 column and eluting the column with PBS. The vesicles were used upon production to minimize dye leakage or aggregation.

### Fluorescence Vesicle Permeability Assay

For the calcein encapsulation experiments, 50 mm of calcein dye was included in the polymer film hydration solution. After production of the calcein‐containing polymersomes, the polymersomes were passed through a size exclusion column to remove unencapsulated calcein. This involved using a Sephadex G‐50 column, eluting in sucrose (500 mm sucrose, PBS). The polymersomes were used upon production to minimize dye leakage or aggregation. Calcein leakage was assessed by fluorescence spectroscopy, with the calcein fluorescence emission recorded at λex/em = 494/514 nm on a Cary Eclipse Fluorometer (Agilent Technologies, USA). Following the measurements, 10% (v/v) Triton X‐100 was added to each solution to induce vesicle lysis and determine the maximum fluorescence intensity of each well. The vesicles were then incubated for 30 min to ensure complete lysis had occurred before the final fluorescence readings were taken ($F_{\max(triton)}$). To analyse the kinetic measurement data, the data at each timepoint (*F_I_*) was normalized to the maximum fluorescence intensity to calculate the release percentage (Equation 1).

(1) $$Release\;\%=\frac{F_{I}}{F_{\max\left(triton\right)}}*100$$

The normalized fluorescence used to compare the different polymersome and vesicle release values at different temperatures was calculated by dividing the fluorescence values (*F_I_*) by the fluorescence intensity at 25 °C ($F_{I(25\;\;C)}$) (Equation 2).

(2)

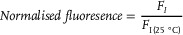




### Polymeric Droplet Formation

A 5 mg mL^−1^ dispersion of nanoscale PEO‐PBO vesicle, prepared accordingly to the protocol described in *Production of PEO‐PBO Polymersomes*, was placed into a PDMS well (≈50 µL total volume) on a microscope slide. The well was sealed with a glass cover slip. The assembled chamber was then placed on top of a Linkam PE120 heating/cooling stage, equipped with an EHEIM water circulation pump system. The sample was heated at a controlled rate of 5 °C min^−1^ until reaching 70 °C. After a 5‐minute incubation at this temperature, the system was rapidly cooled back to room temperature.

### Optical Microscopy

A Nikon eclipse Ti2‐U inverted microscope with a CoolLED pE‐300^white^ and a Nikon DS‐Qi2 camera was used to image the polymeric droplets. These solutions were imaged by filling a PDMS well on a microscope slide, a cover slip was added on top of the well to seal the sample chamber. For fluorescence imaging a TRITIC filter cube was used to visualize the rhodamine dye, a Cy5 filter cube was used to image the Nile Red or cy5.5 dye and a GFP filter cube was used to image the GFP‐expressing bacteria and calcein dye.

For the calcein permeation assay, 0.1 µL of calcein (0.2 mm in PBS) was added to the sample chamber (≈40 µL of sample) and thoroughly mixed with a pipette.

For experiments involving temperature, a Linkam PE120 Peltier heating stage with an EHEIM professional 4+ water filter unit was placed on the microscope. For experiments involving temperature, the samples were subjected to a heating rate of 1 °C min^−1^.

For the confocal microscopy experiments, a Leica stellaris 8 inverted microscope was used with a 20x/0.75 NA objective and a 543 nm excitation laser. Emission was recorded between 458–750 nm with a scan speed of 400 Hz.

The polydispersity index of the polymeric droplets was calculated using the following equation.

σ represented the standard deviation and *d* was the mean of the population.^[^ ^83^ ^]^

(3) $$Polydispersity={\left(\frac{\sigma }{d}\right)}^{2}$$

The fluorescence recovery after photobleaching experiments was performed by photobleaching the Nile red dye using 100% laser power for the bleaching time. The fluorescence was then normalized using the below equation (Equation 4) where *F_max_* is the maximum fluorescence signal before bleaching and *F_t_* is the fluorescence signal at a given time point. The line profile results were also normalised using the same equation except F*_max_* was the maximum fluorescence intensity on the line profile and F*_t_* was a position on the line profile.

(4) $$Normalised\;fluorescence=\frac{F_{t}}{F_{max}}$$

To fit the polymeric droplet contractility data the following logistic equation was used where *x* is the temperature at a given time point, *A* + *B* are the maximum diameter, *k* is the steepness of the contraction, *x*_0_ is the temperature where the polymeric droplets have undergone a 50% contraction and *B* is the minimum diameter (the offset of the function).

(5) $$Contractility\;fit=\frac{A}{1+e^{-k\left(x-x_{0}\right)}}+B$$

### Laurdan Assay

Laurdan‐labeled SUVs (0.5 mol%) were made using extrusion. A Horiba Yvon Fluoromax 4 fluorimeter was connected to a temperature control block. Fluorescence wavelength‐corrected emission spectrum from 400 to 600 nm of 1 mg mL Laurdan‐labelled SUVs was recorded with an excitation wavelength of 360 nm and the fluorescence intensity at each temperature was normalized to 0–1. Generalized polarization (GP) values were calculated using Equation 6 where *I* is the measured intensity followed by a number indicating the wavelength of I.

(6) $$General\;Polarization\;\left(GP\right)\;function=\frac{I435\;-\;I490}{I435+I490}$$

### Preparation of Giant Unilamellar Vesicles with Nested Polymersomes

A DOPC lipid film of 2 mg was produced by dissolving DOPC in chloroform before removing the chloroform under a gentle stream of N_2_. The film was then dried overnight under vacuum to remove the residual chloroform. The dried film was then dissolved in 1 ml of mineral oil to produce a 2 mg mL^−1^ lipid in oil solution. To aid this process the lipid in oil solution was sonicated for 30 min at 50 °C.

An emulsion was then created by adding 20 µL of PEO‐PBO polymersomes in PBS pH 7.4 (see *Production of PEO‐PBO Polymersomes*) and 0.5 m sucrose in PBS to 200 µL of the lipid‐containing mineral oil. This emulsion was then vortexed for 30 s and pipetted up and down 10 times to ensure the aqueous droplets were well dispersed and coated with a lipid monolayer. This emulsion was then layered on top of 200 µL of 0.5 m glucose in PBS contained in an Eppendorf to produce a column. The column was then centrifuged at 9000 g for 10 min to produce a pellet containing the DOPC GUVs with the nested PEO‐PBO polymersomes. The supernatant was then removed, and the pellet was then resuspended in fresh 0.5 m glucose in PBS ready for imaging.

### Preparation and Trapping of Bacteria

Escherichia coli BW25113 bacterial cells expressing GFP and used in the entrapment assay were cultured in LB medium at 37 °C with shaking at 220 rpm. Cells were then transferred to fresh LB medium at a final dilution of 10^−7^ and grown to an OD of 0.2–0.3 before being placed with the PEO‐PBO polymersomes. The bacteria were trapped by subjecting the solution to two heating/cooling cycles from 25 to 70 °C at a rate of 20 °C min^−1^.

### Statistical Analysis

Where appropriate experimental data was statistically analyzed, and the results were shown as the mean ± standard deviation, the number of replicates are given in the figure captions of the respective figure.

## Conflict of Interest

The authors declare no conflict of interest.

## Author Contributions

M.E.A. and Y.S. contributed equally to this work. M.E.A and Y.S. helped to perform and analyze the data and wrote the manuscript; C.L.C. performed the SAXS and polarizing microscopy experiments; M.P.P. supervised the Laurdan assay experiment; Y.E and O.C. hosted this research and helped with the final version of the manuscript; C.C designed and performed experiments, analyzed the data and revised the manuscript.

## Supporting information



Supporting Information

Supplemental Video 1

Supplemental Video 2

Supplemental Video 3

Supplemental Video 4

Supplemental Video 5

## Data Availability

The data that support the findings of this study are available from the corresponding author upon reasonable request.
