# Supplementary material for: Thermally Driven Dynamic Behaviors in Polymeric Vesicles
Source: Small. 2025 Mar 5;21(33):2411220. doi: 10.1002/smll.202411220 (PMC12372441; doi:10.1002/smll.202411220)
Supplement: Supplementary file 1 — Supporting Information [file SMLL-21-2411220-s003.docx]

Supporting Information

Thermally Driven Dynamic Behaviors in Polymeric Vesicles

Matthew E. Allen^1,2,3,4†^, Yeyang Sun^1,4,5†^, Chi Long Chan^1^, Miguel Paez-Perez^1,4^, Oscar Ces^1,2,4*^, Yuval Elani^3,4*^, Claudia Contini^4,5*^

^1^Department of Chemistry, Molecular Sciences Research Hub, Imperial College London, London, W12 0BZ, UK

^2^Institute of Chemical Biology, Molecular Sciences Research Hub, Imperial College London, London, W12 0BZ, UK

^3^Department of Chemical Engineering, Imperial College London, South Kensington, London SW7 2AZ, UK

^4^fabriCELL, Molecular Sciences Research Hub, Imperial College London, London, W12 0BZ, UK

^5^Department of Life Sciences, Imperial College London, South Kensington, London SW7 2AZ, UK

† These authors contributed equally

E-mail: [c.contini@imperial.ac.uk](mailto:c.contini@imperial.ac.uk), [y.elani@imperial.ac.uk](mailto:y.elani@imperial.ac.uk), [o.ces@imperial.ac.uk](mailto:o.ces@imperial.ac.uk)

**Supporting figures**


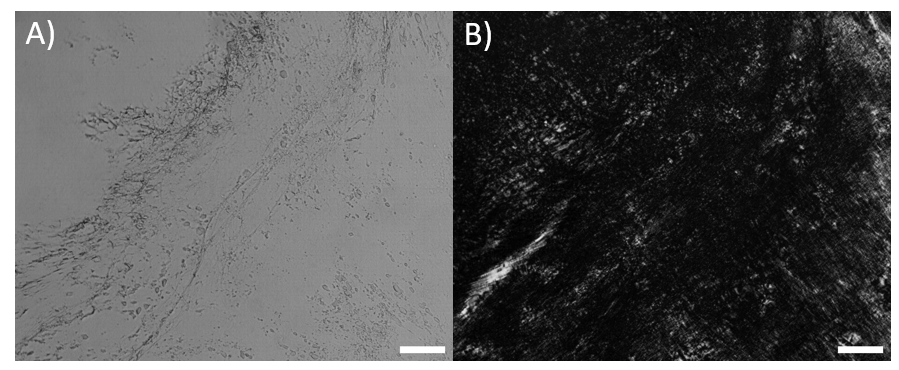


**Figure S1:** **Polarising microscopy of the aqueous PEO-PBO di-block copolymer dispersion.** Panel A shows the brightfield while panel B is the texture under polarisers. Upon viewing through polarisers (panel B), limited birefringence was seen which is characteristic of a sponge phase^[1]^. Upon compression the sample flowed demonstrating fluidity. The scale bar is 100 µm.


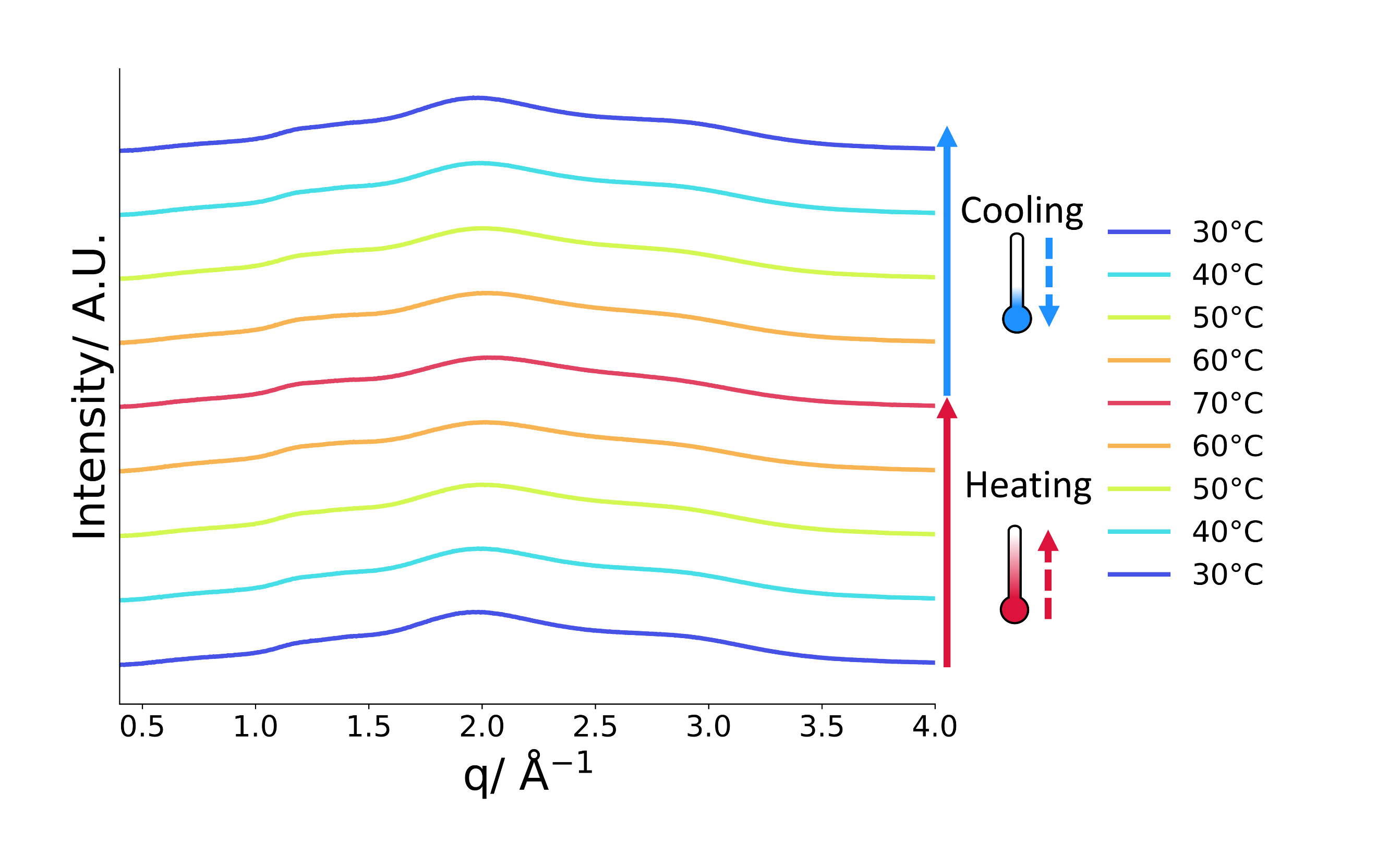


**Figure S2: Wide angle scattering of an aqueous PEO-PBO di-block copolymer dispersion.** The thermometers indicate the heating and cooling sections of the WAXS spectra. Upon both heating to 70°C and cooling back to 25°C little change in the WAXS spectra was seen indicating no gel to fluid transitions were occurring. Furthermore the broad peaks are indicative of fluid sample^[2]^, showing that the polymer exists in a liquid rubbery state.


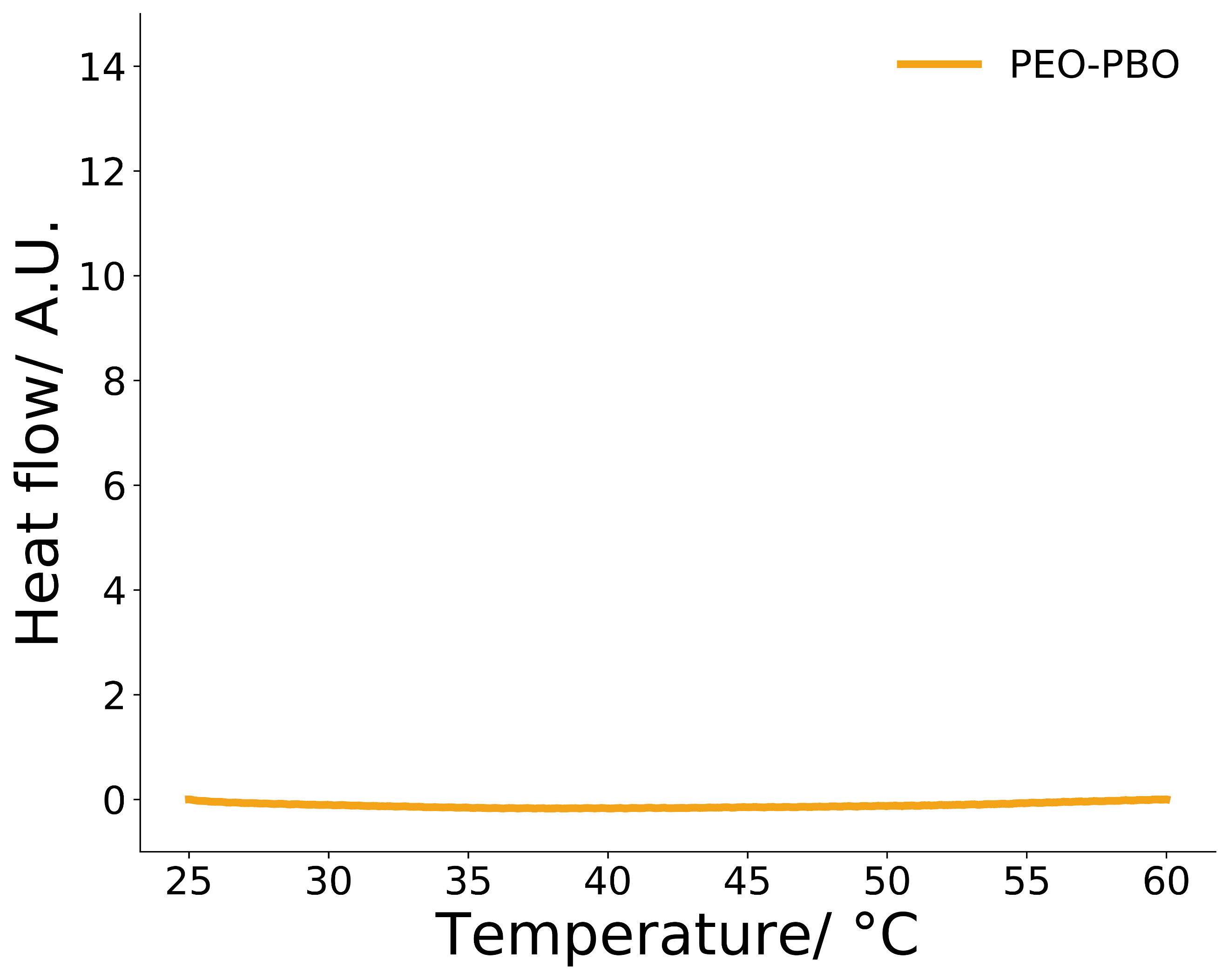


**Figure S3: Differential scanning calorimetry of the PEO-PBO polymer.** Upon heating from 25 to 60°C no phase transition was observed. For similar polymer systems, the glass transition temperature (T_g_) falls at a lower temperature than this range^[3]^. As a consequence, the PEO-PBO polymer stays in a liquid rubbery state across the temperature range shown. Thus, the reported changes in the temperature-driven properties are not due to a shift in the polymer phase. This matches the data obtained from the WAXS spectra.


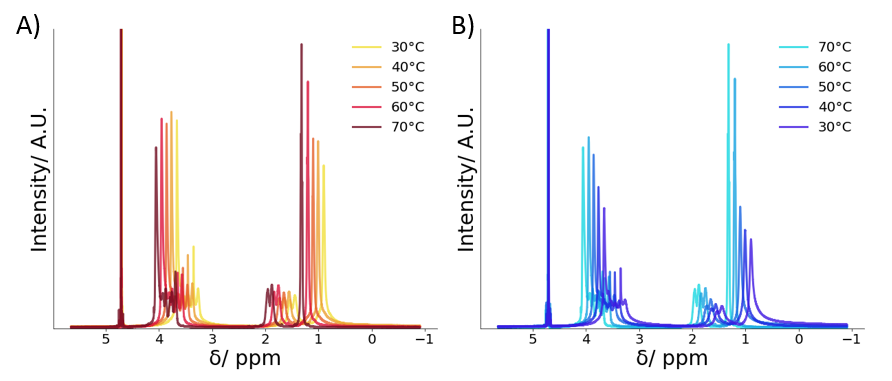


**Figure S4: Heating and cooling ^1^H NMR intensity plots of the PEO-PBO copolymer. A)** An intensity plot showing that upon heating the NMR signals around 1 and 2 ppm become stronger while the signal at 4 ppm decreases in intensity. **B)** A cooling NMR intensity plot demonstrating that the signals at 1, 2 and 4 ppm become weaker upon cooling. The intensity change in the signals at 1 and 2 ppm is reversible while the 4 ppm signal is not. This indicates that upon heating and cooling polymer chain mobility and polymeric surface curvature is altered.

**
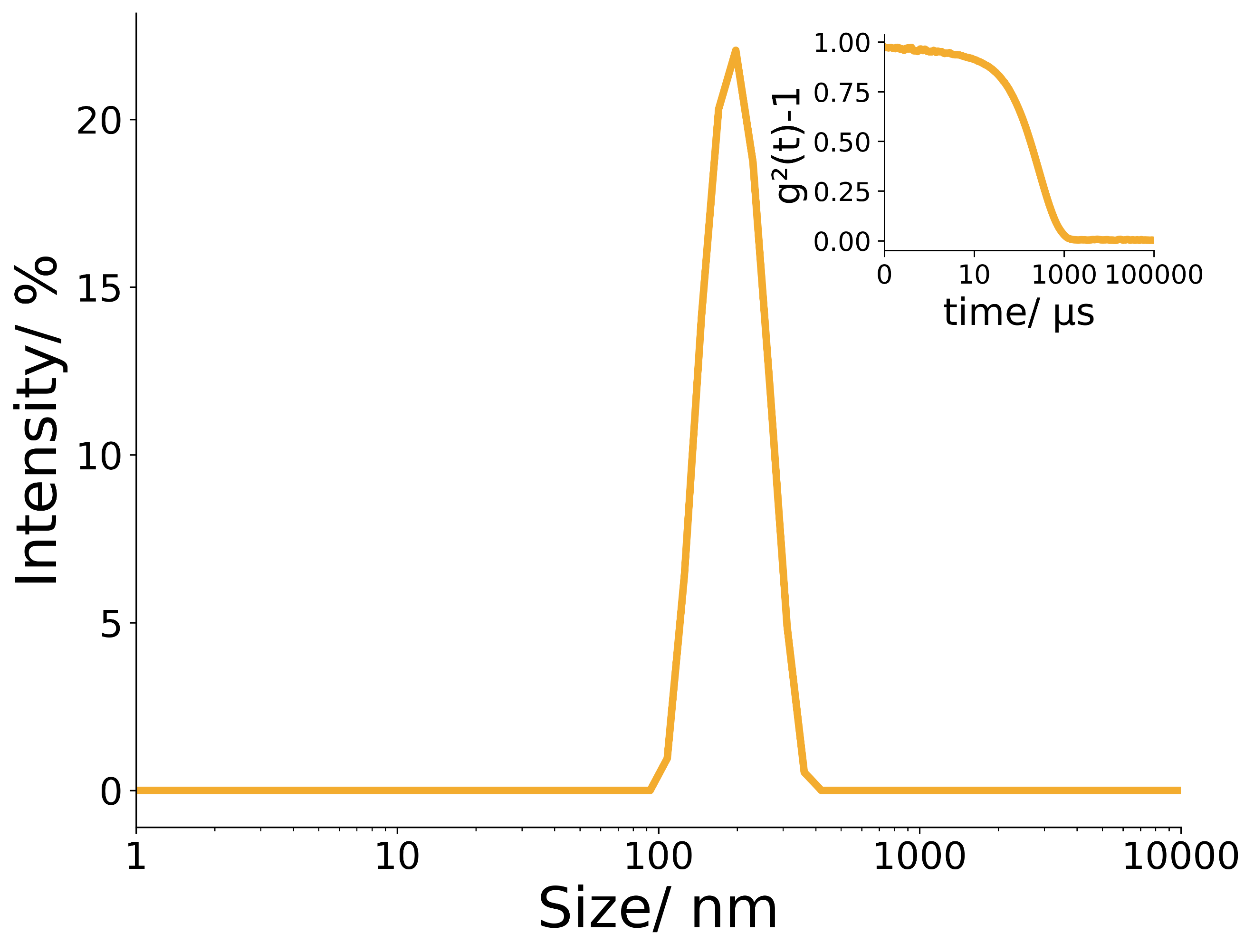
**

**Figure S5: Dynamic light scattering of PEO-PBO polymersomes at 25°C.** DLS size distribution and corresponding autocorrelation function of a population of PEO-PBO polymersomes. The mean diameter was 115 nm with a smooth autocorrelation function. This demonstrates the production of a clear single population of polymersomes.


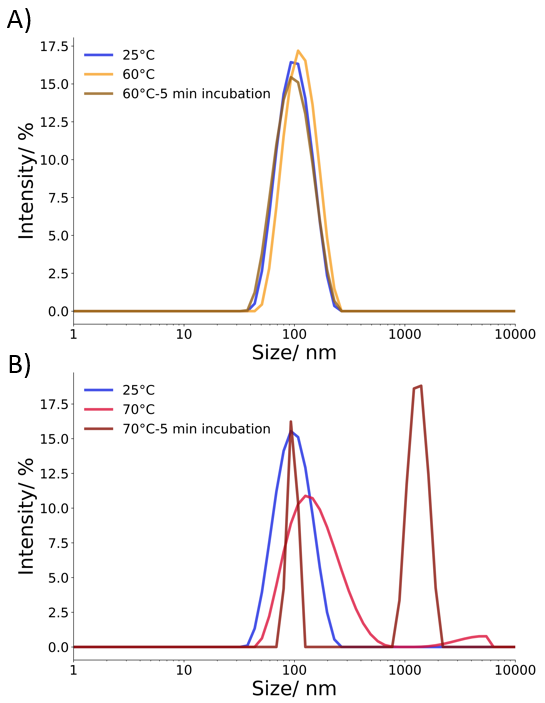


**Figure S6: DLS incubation scans of PEO-PBO polymersomes.** The polymersomes were heated up to 60 °C (A) or 70 °C (B) and incubated at each temperature for 5 minutes. At 60 °C a limited change in size was observed while at 70 °C a larger change in size was observed which became more prominent after 5 minutes. This shows that temperature drives the formation of larger polymeric structures.


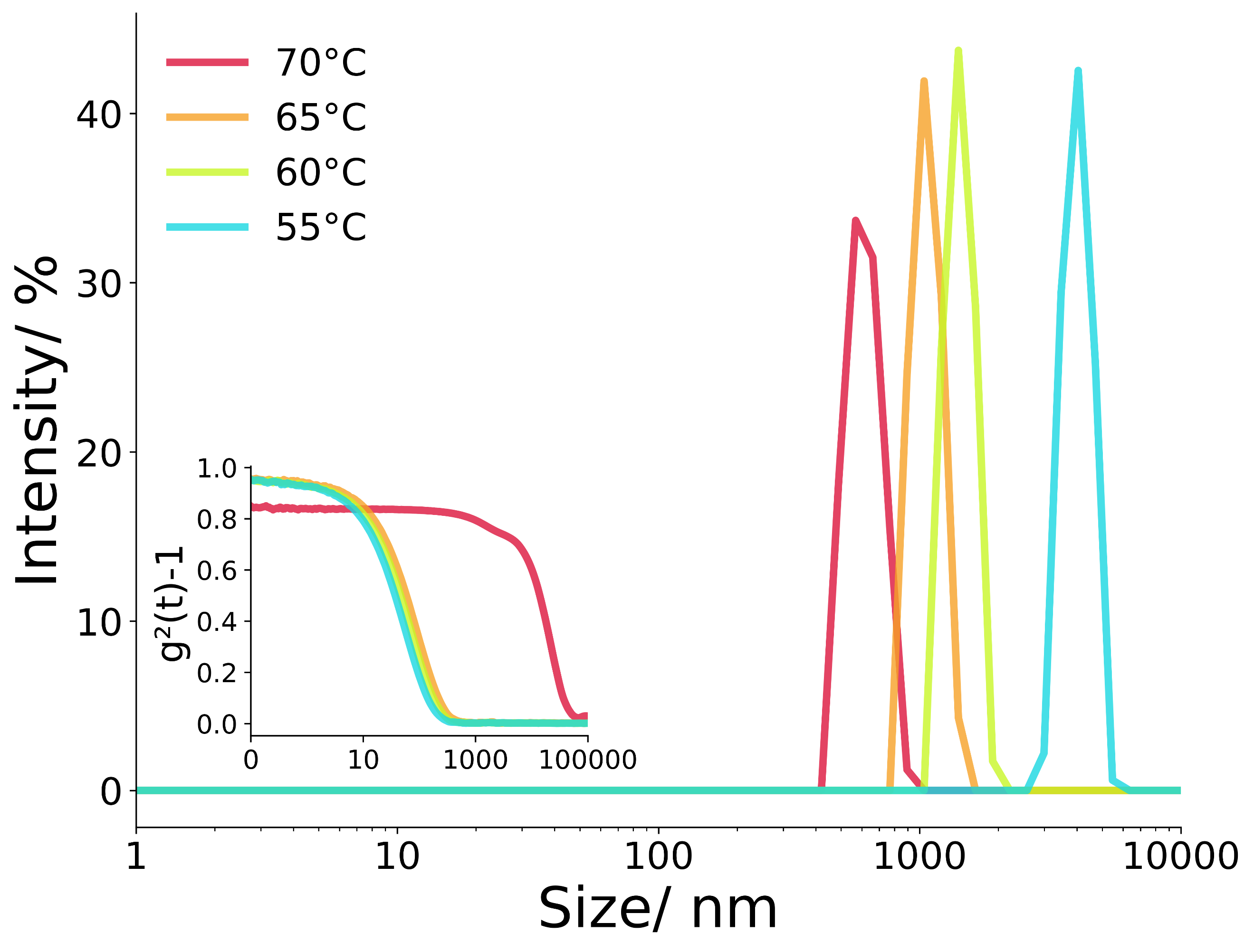


**Figure S7: DLS cooling scans of PEO-PBO polymersomes.** Upon cooling from 70°C to 55°C a further increase in size was seen. Meanwhile the autocorrelation function reverts back to its original shape upon cooling. These two pieces of data demonstrate some population reversibility is observed upon cooling (the autocorrelation function) although larger size structures are still present.


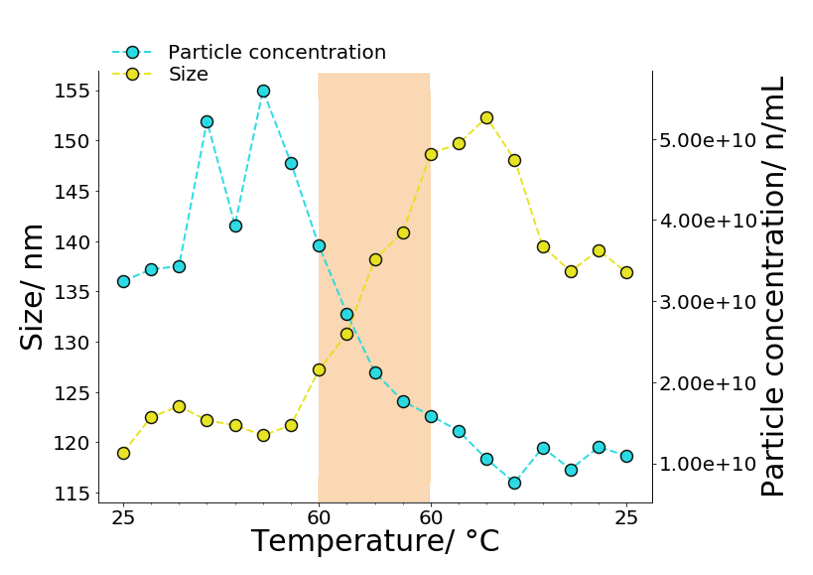


**Figure S8: Particle concentration vs size plot of 1 DLS heating/ cooling cycle.** The shaded region indicates the temperature being held at 60°C in the heating/ cooling cycle. Within this cycle, an increase in particle size and a decrease in particle concentration was seen. This further supports that the polymersomes are forming larger structures consisting of multiple polymersomes at elevated temperatures.


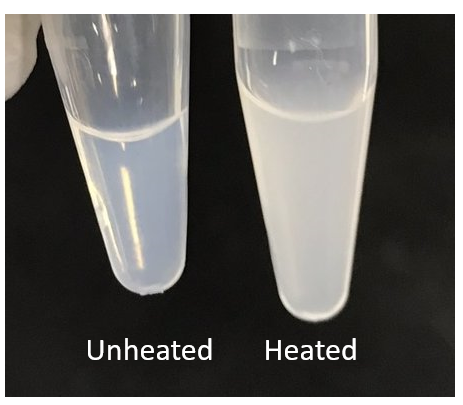


**Figure S9: Images of heated and unheated PEO-PBO polymersome solutions.** On heating a clear increase in turbidity can be seen indicating the formation of larger particles.

**
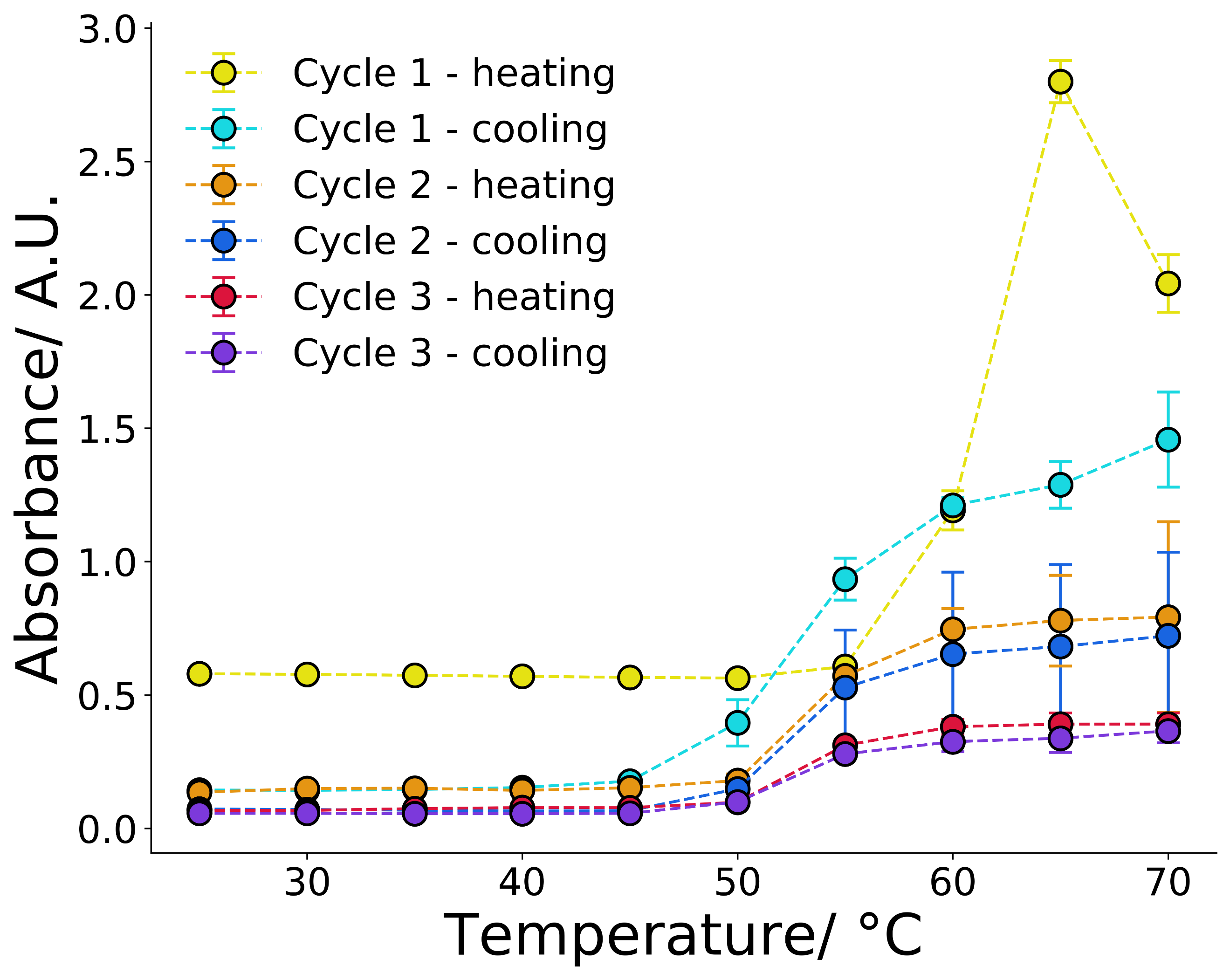
**

**Figure S10: Turbidity heating cycles of PEO-PBO polymersomes.** Upon heating of the PEO-PBO polymersomes an increase in absorbance is seen above 50°C, this is consistent with the formation of larger particles. Upon cooling the absorbance decreases demonstrating some reversibility to the larger particle formation. This behaviour is repeated over 3 cycles, with each cycle the absorbance increase becomes less significant showing that there is some irreversibility present upon larger particle formation. This matches DLS observations seen within figures 3C and 3D. The error bar is from n=3 samples.

**
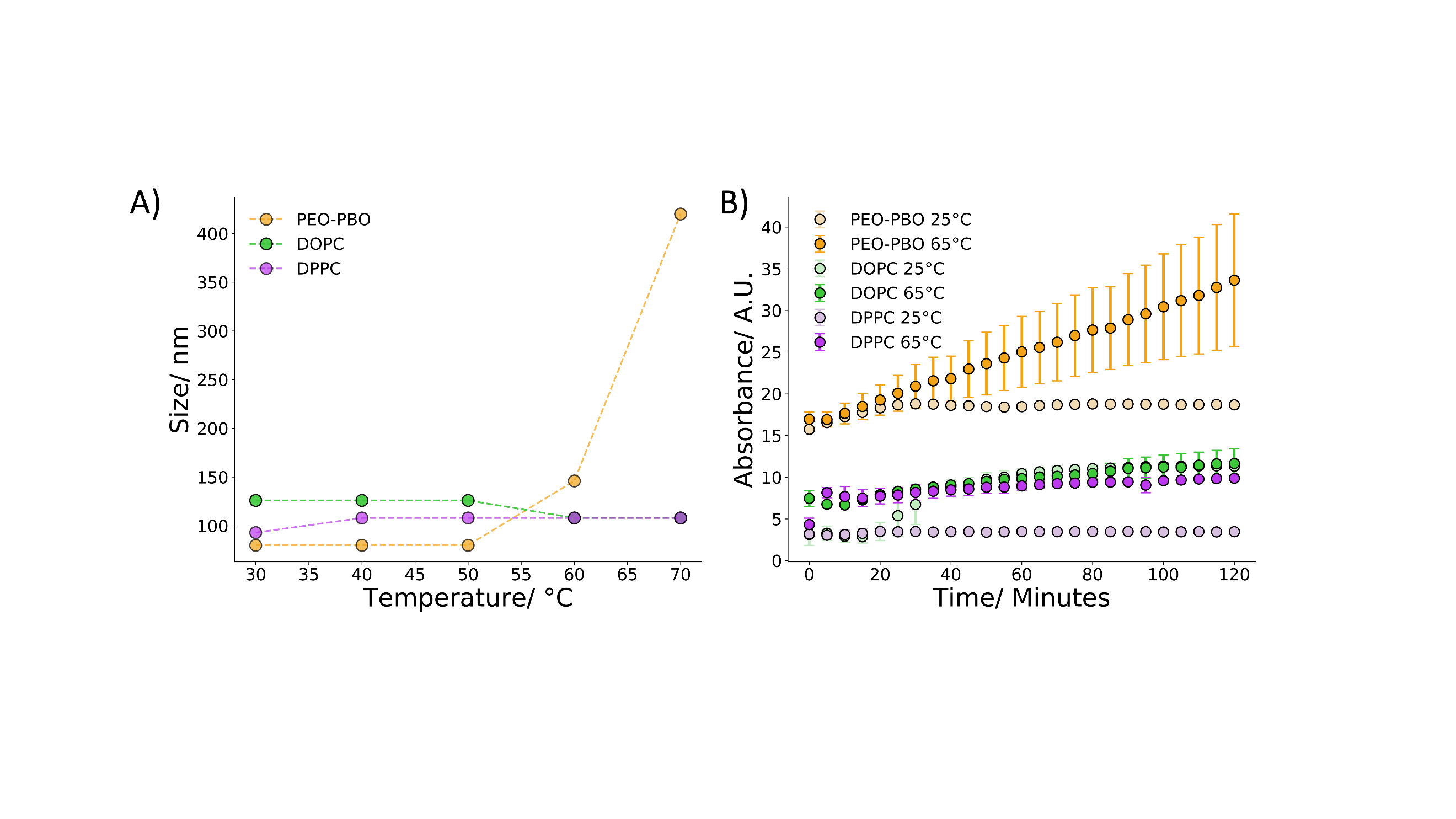
**

**Figure S11: Comparison of the effects of temperature on PEO-PBO polymersomes vs lipid vesicles. A)** DLS scans showing how PEO-PBO polymersomes respond to temperature compared to lipid vesicles. Upon heating PEO-PBO polymersomes extruded to 100 nm demonstrate an increase in size from around 60°C. For thermoresponsive (DPPC) and non thermoresponsive (DOPC) vesicles extruded to 100 nm no increase in size was evident. **B)** A turbidity assay indicating that heated PEO-PBO polymersomes have a significantly difference absorbance compared to 25°C polymersomes. The thermoresponsive (DPPC) and non thermoresponsive (DOPC) lipid vesicles do not show a significantly difference absorbance upon heating. The results show that the polymer membrane exhibits a different thermal response to vesicle systems. The error bars on the turbidity assay are from n=3 samples.


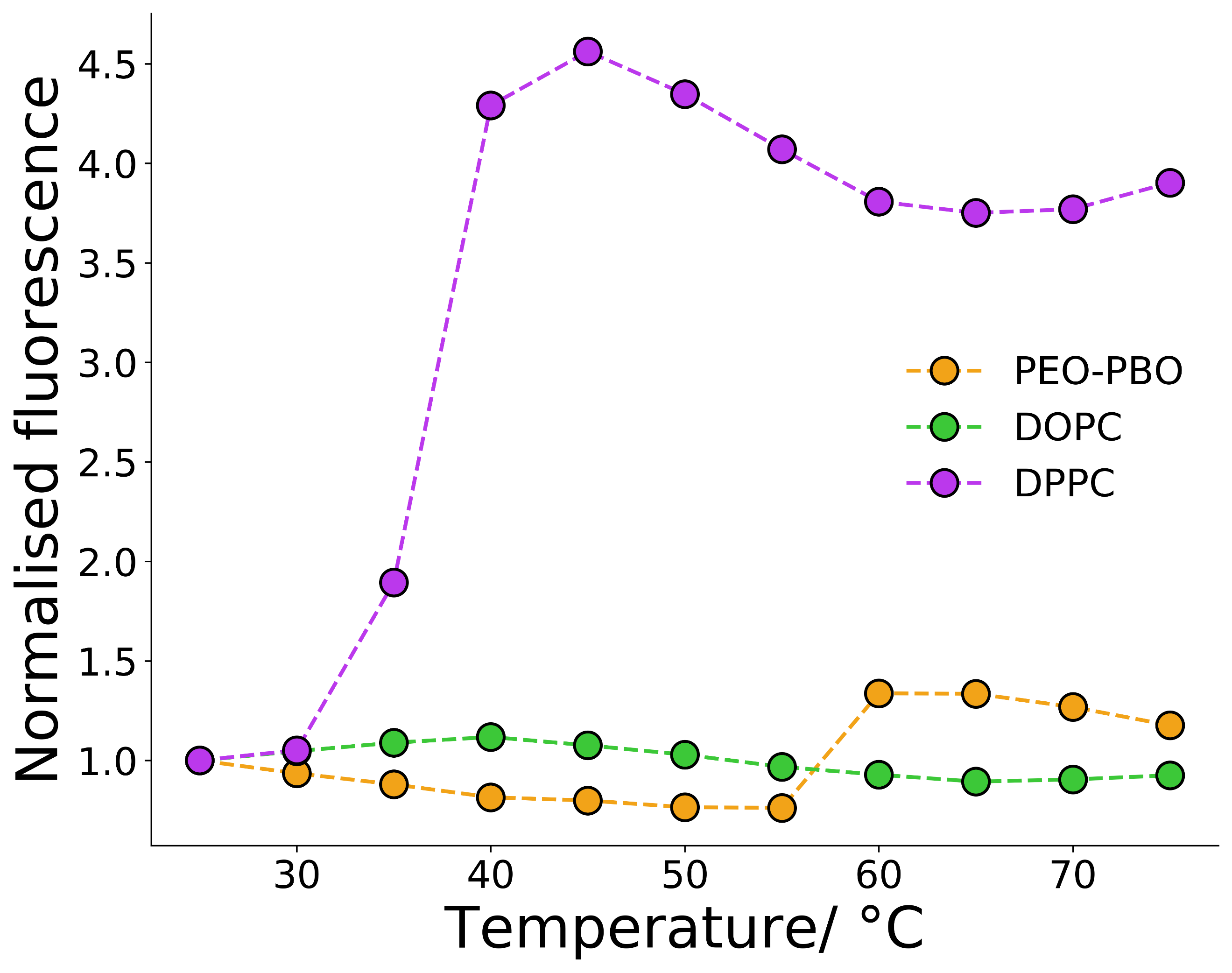


**Figure S12: Normalised temperature gradient calcein release of vesicles and polymersomes.** Through measuring the fluorescence at 5°C temperature intervals it can be seen that DOPC vesicles containing no thermoresponsive components do not release calcein while the thermoresponsive DPPC vesicles do release calcein (shown by the increase in fluorescence). The PEO-PBO polymersomes also show calcein release at 60°C although the scale of release is smaller than the DPPC vesicles. The decrease in fluorescence signal is due to photobleaching. The normalisation was performed by normalising all values to the fluorescence at 25°C.


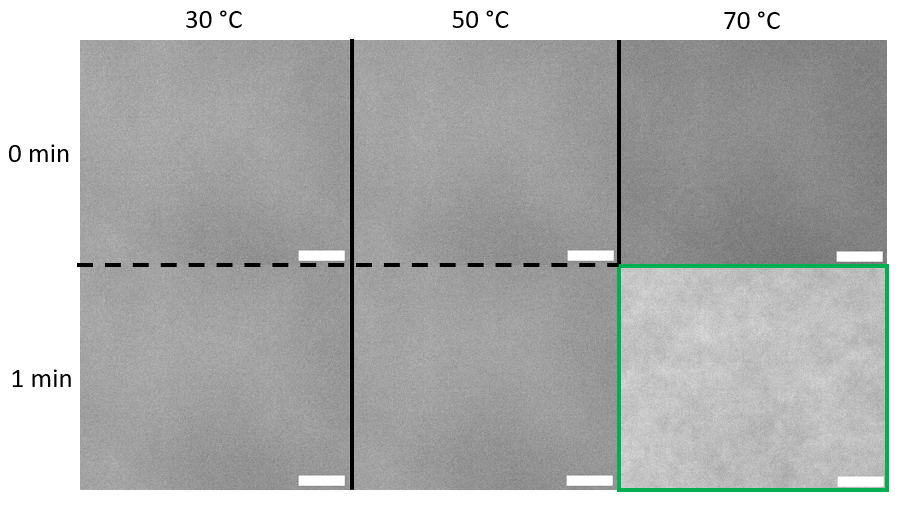


**Figure S13: Incubating PEO-PBO vesicles at constant temperatures**. A collection of brightfield microscopy images of PEO-PBO vesicles incubated at a range of different temperatures. After 1 minute there is no change in optical appearance apart from at 70 ºC. This shows that the vesicles only begin to fuse at temperatures above the transition temperature. The scale bars are 20 μm.


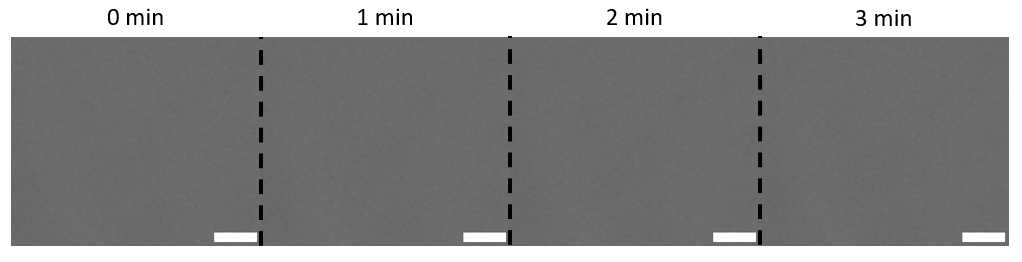


**Figure S14: Incubating PEO-PBO vesicles at 45 ºC for extended time periods**. A set of brightfield microscopy images of PEO-PBO vesicles incubated at 45 ºC for 3 minutes. There is no change in optical texture demonstrating that the vesicles are stable at this temperature and not forming larger structures. The scale bars are 20 μm.


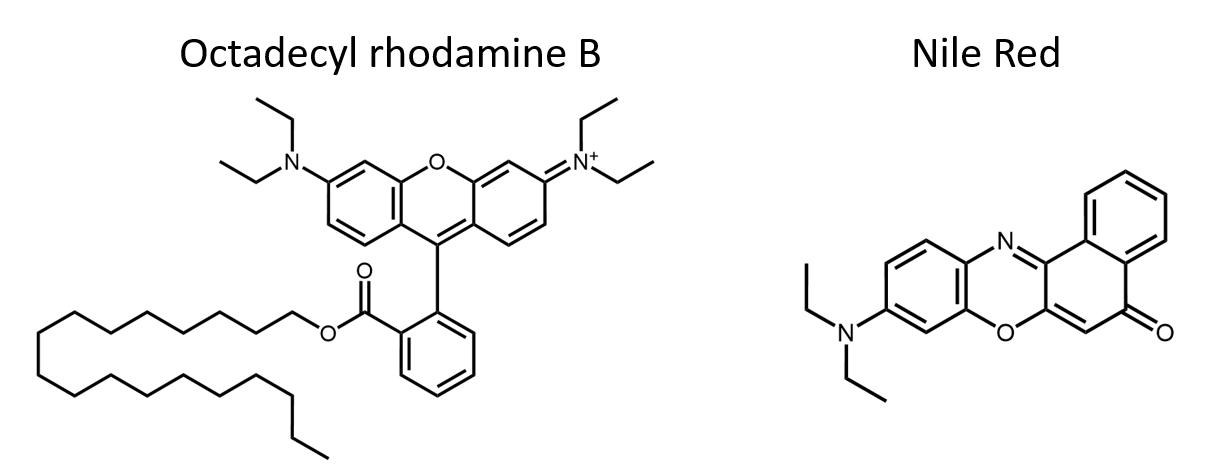


**Figure S15: Chemical structures of the fluorescent lipophilic dyes utilised.**


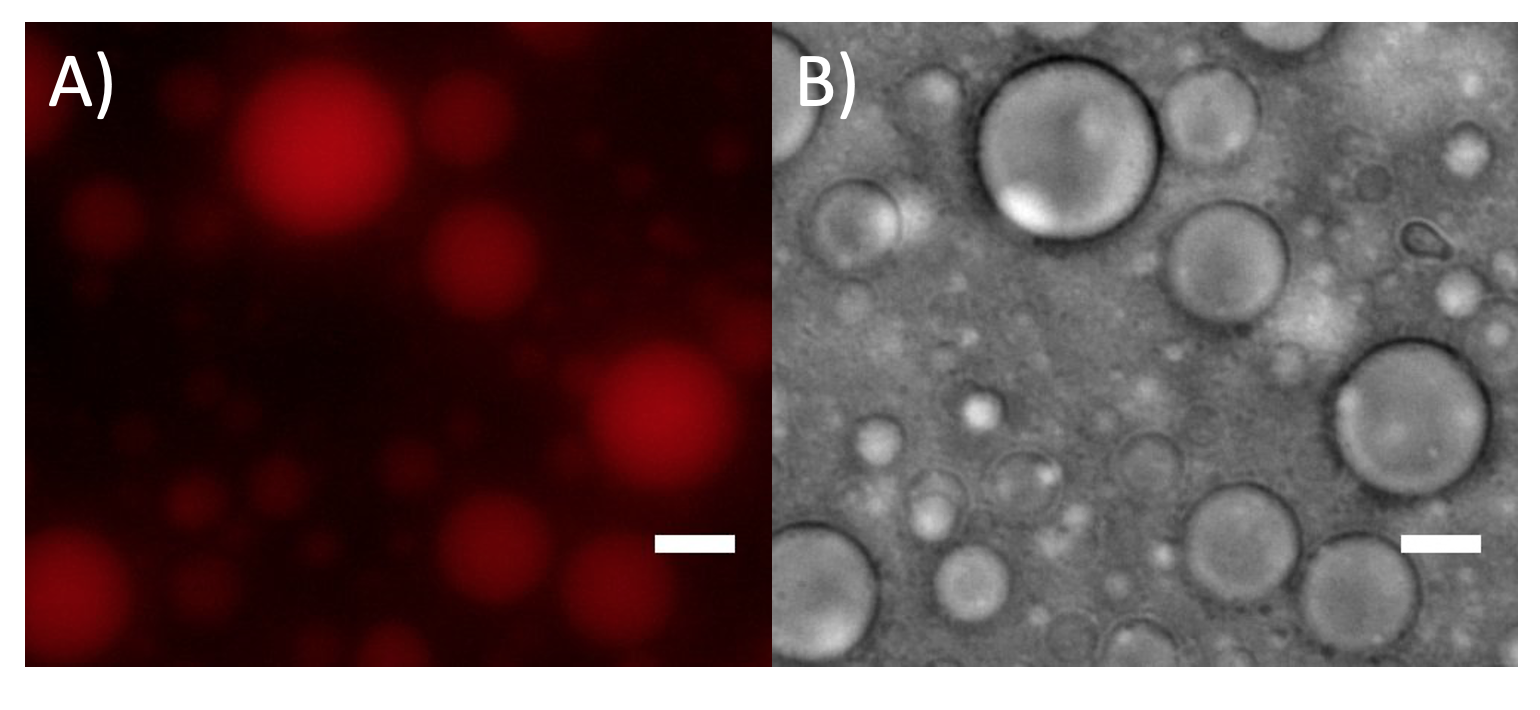


**Figure S16: Population images of polymeric droplets stained with rhodamine B.** Fluorescence (panel A) and brightfield (panel B) images of a population of spherical polymeric droplets stained with a rhodamine B dye. The fluorescence is localised to the droplets. The scale bars are 20 µm.


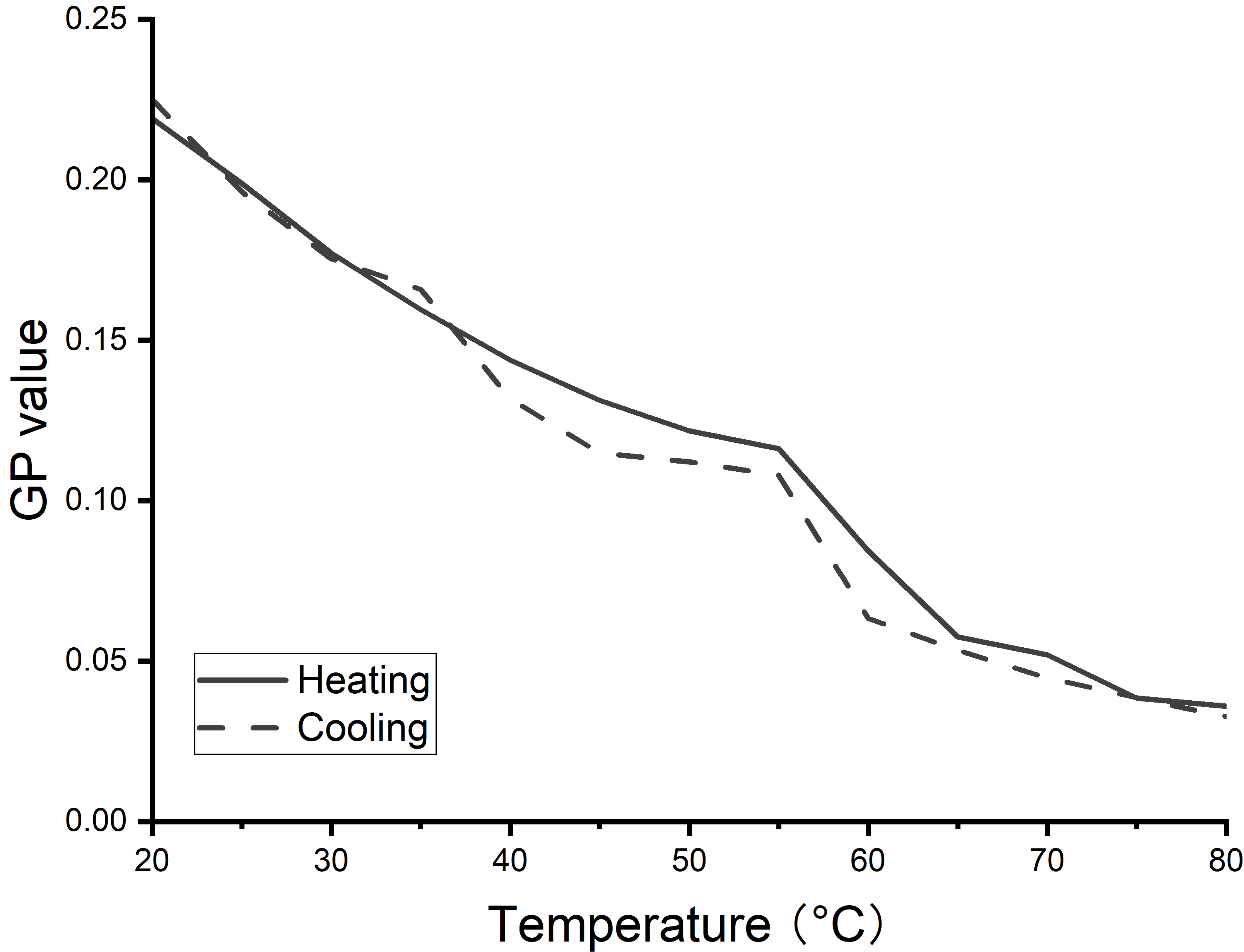


**Figure S17: Temperature responsiveness of Laurdan dye (excitation = 360 nm).** The general polarisation (GP) value changes on heating and cooling, showing that the Laurdan dye can access water and undergo dipolar relaxation more easily at higher temperatures.


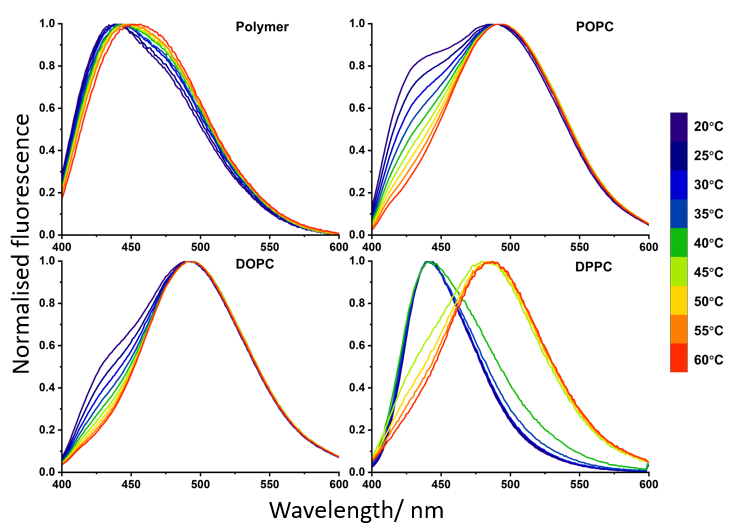


**Figure S18: Laurdan comparison between the polymer and lipids.** The polymer can be seen to possess a gradual spectral change in maximum emission. This behaviour is different to lipids which are fluid in this temperature interval and have no emission maxima spectral shift (DOPC and POPC) or DPPC lipids which have a large spectral shift upon passing through their gel to fluid transition temperature. This highlights that the polymer undergoes a different thermoresponsive transformation to the lipids due to the different structural dynamics between the systems.


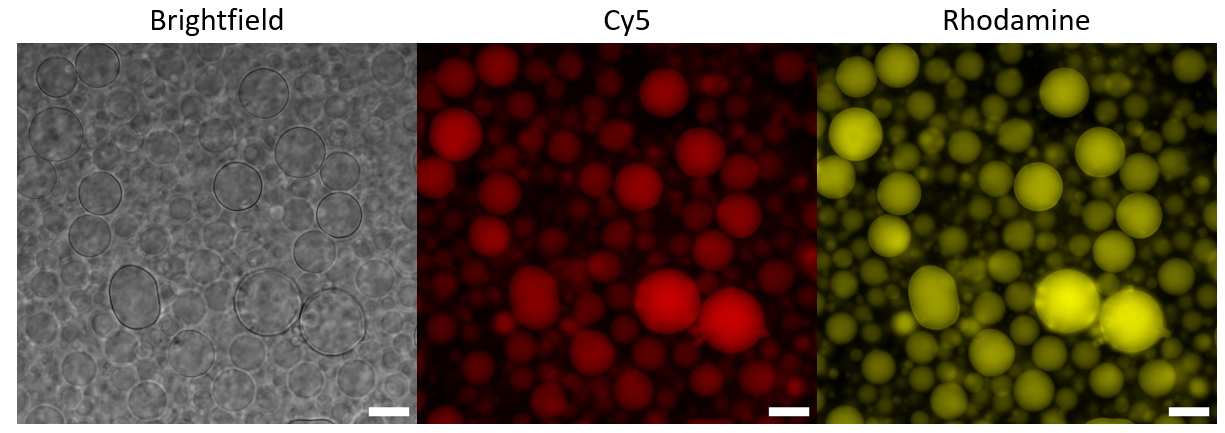


**Figure S19: Formation of polymeric droplets using different polymersome populations.** Polymeric droplets were formed from two polymersome populations incubated together. One population contained a Cy5 dye and the other contained a Rhodamine dye. The microscopy images of the produced droplets showed all droplets had even fluorescent signals from both fluorophores, indicating that both populations were fusing together to produce the polymeric droplets. The scale bars are all 50 µm.


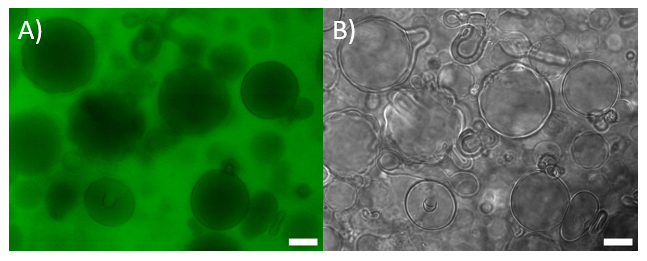


**Figure S20: Population images of calcein permeation into polymeric droplets.** A fluorescence (panel A) and brightfield (panel B) image of the addition of calcein to the polymeric droplet solution. The darker regions in the fluorescence channel correspond to the positions of the droplets and thus indicate limited calcein permeation into their structure demonstrating that the produced polymeric membrane separates the internal and external aqueous solutions successfully. The scale bars are 20 µm.


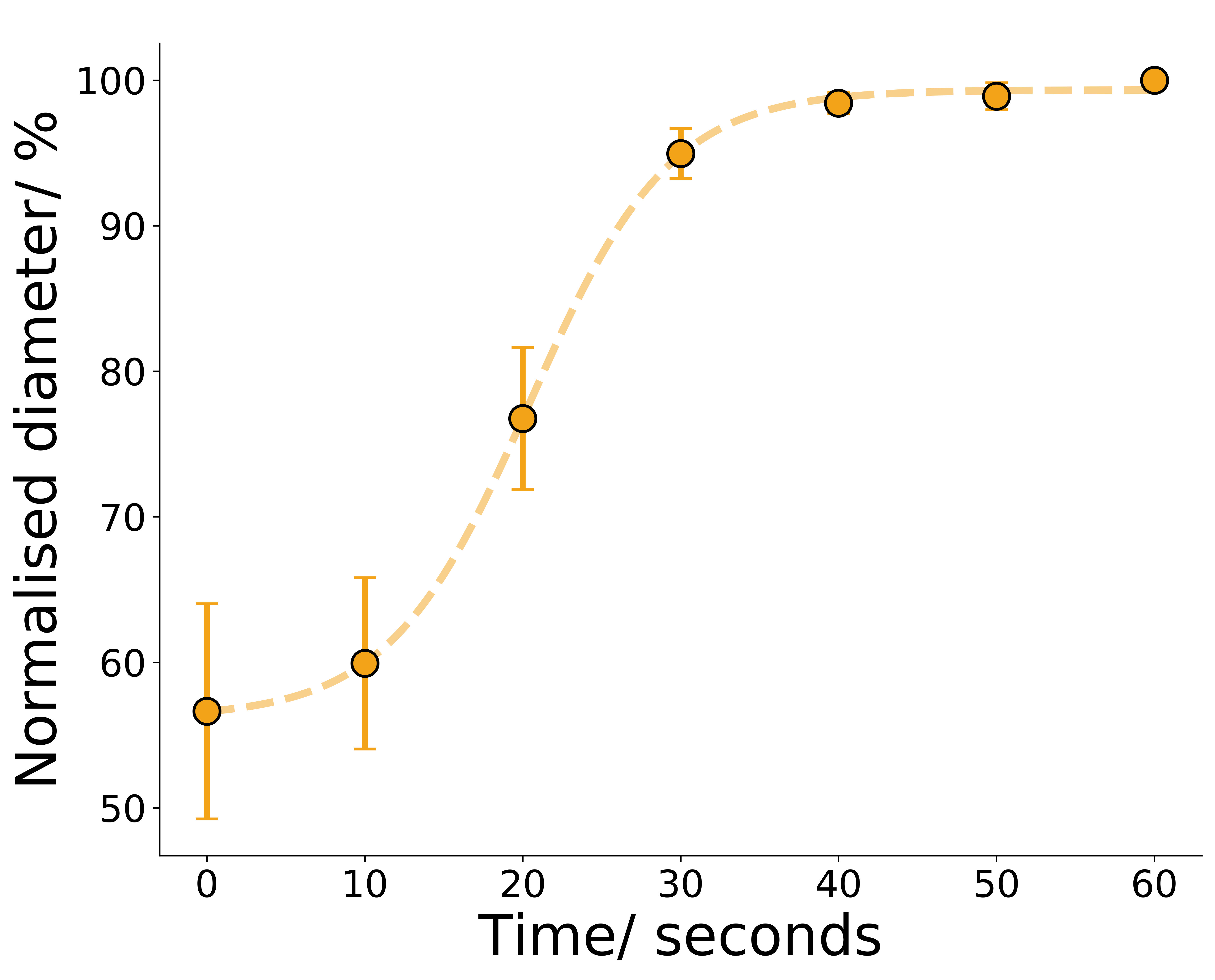


**Figure S21: Dynamics of the size change within polymeric droplets.** Polymeric droplets heated to 70 °C were left to cool to room temperature with no control over the cooling rate. Within 40 seconds the crumpled polymeric droplets had cooled enough to revert back to their original size with a 35% change in size occurring over a 20 second period. This shows that the contractility operates on a timescale of seconds. The error bars were obtained from the standard deviation of n=3 PEO-PBO polymeric droplets.


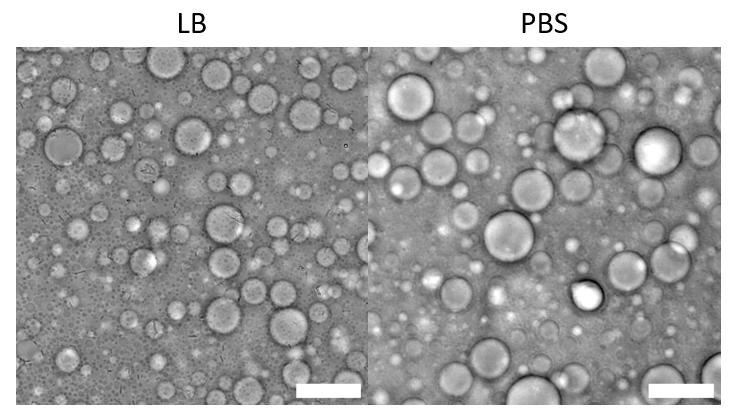


**Figure S22: Comparison of polymeric droplets formed in LB and PBS.** Microscopy images of Polymeric droplets formed in LB containing bacteria and PBS. The polymeric droplets in both conditions had a similar appearance, demonstrating that the polymeric droplets can be formed and are stable in a range of different conditions. The scale bars are 50 µm.


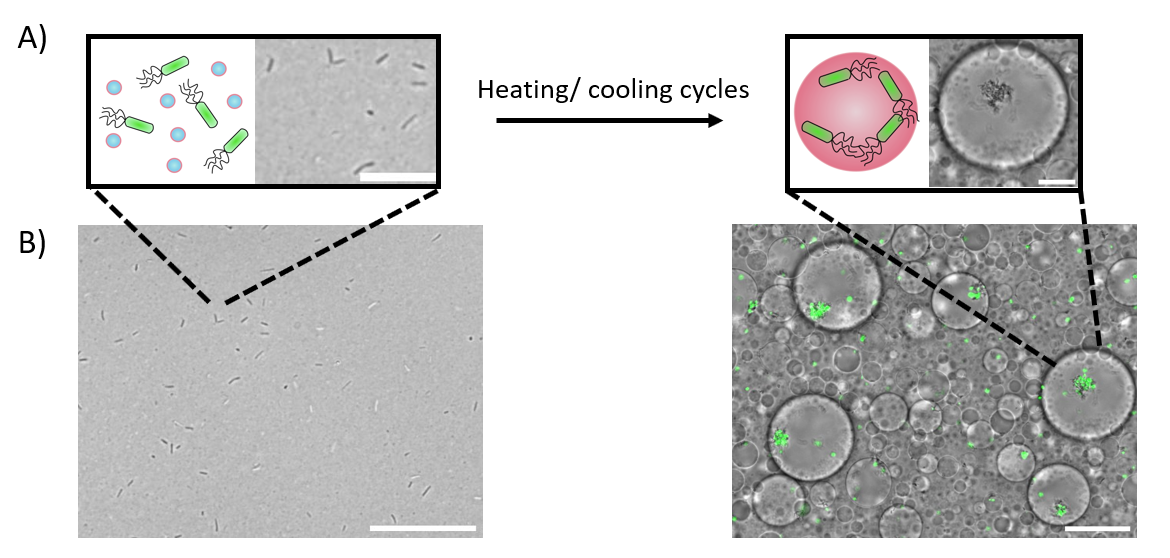


**Figure S23: Trapping of bacteria in polymeric droplets in LB containing solutions. A)** Illustrations with accompanying brightfield images showing how PEO-PBO polymersomes can trap bacteria in LB solutions. The scale bars are 20 µm. **B)** Population images of GFP expressing bacteria before and after trapping in polymeric droplets. Prior to the heating and cooling cycles, the bacteria are present throughout the LB solution while after the bacteria are localised within the produced polymer droplets. The green fluorescence overlay in the after image shows the positioning of the bacteria. The scale bars are 50 µm.

**SI video descriptions**

**Video S1-** Recovery of fluorescence in a polymeric droplet that had been photobleached. The scale bar is 10 µm.

**Video S2-** PEO-PBO polymeric synthetic cells shrinking upon heating to 70°C. The scale bar is 20 µm.

**Video S3-** Crumpled PEO-PBO polymeric synthetic cells expanding upon cooling to 25°C. The scale bar is 20 µm.

**Video S4-** Fusion of two PEO-PBO polymeric synthetic cells at 55°C. The scale bar is 20 µm.

**Video S5-** Expansion of a fused PEO-PBO polymeric synthetic cell upon cooling to 25°C. The scale bar is 20 µm.

**References**

[1] S. H. Bhansali, A. S. Malik, J. M. Jarvis, I. Akartuna, D. M. Dabbs, J. D. Carbeck, I. A. Aksay, *Langmuir* **2006**, *22*, 4060.

[2] D. Lamba, in *Encycl. Membr.*, Springer, Berlin, Heidelberg, **2016**, pp. 2040–2042.

[3] N. S. Vrandečić, M. Erceg, M. Jakić, I. Klarić, *Thermochim. Acta* **2010**, *498*, 71.
